# Supplementary material for: The Triterpenoids from Munronia pinnata and Their Anti-Proliferative Effects
Source: Molecules. 2023 Sep 28;28(19):6839. doi: 10.3390/molecules28196839 (PMC10574247; doi:10.3390/molecules28196839)
Supplement: Supplementary file 1 [file molecules-28-06839-s001.zip › molecules-2547909-supplementary.pdf]

The Triterpenoids and Their Anti-proliferative Effects from *Munronia pinnata*

Xuerong Yang[1, #], Peiyuan Liu[1,3,#], Yulu Wei[1], Jingru Song[1], Xiaojie Yan[1], Xiaohua Jiang [1], Jianxing Li[4], Xiangqin Li[4], Dianpeng Li [1,2*], Fenglai Lu [1,*]

[1] Guangxi Key Laboratory of Plant Functional Phytochemicals and Sustainable Utilization, Guangxi Institute of Botany, Guangxi Zhuang Autonomous Region and Chinese Academy of Sciences, No. 85, Yanshan Road, Yanshan District, Guilin 541006, China

[2] Guangxi University of Chinese Medicine, No. 13, Wuhe Road, Nanning 530200, Guangxi Province, China

[3] School of Pharmacy, Guilin Medical University, No. 1 Zhiyuan Road, Lingui District, Guilin 541199, Guangxi, China

[4] Guangxi Key Laboratory of Plant Conservation and Restoration Ecology in Karst Terrain, Guangxi Institute of Botany, Guangxi Zhuang Autonomous Region and Chinese Academy of Sciences, No. 85, Yanshan Road, Yanshan District, Guilin 541006, China#These authors contribute equally to this work and joint first authors.

Contents of Supporting Information

Table S1. Partial $^{3}J_{H,H}$ and $^{2,3}J_{C,H}$ data for munropenes A-F (**1**-**6**)..4
Figure S1. Possible biogenetic pathway of munropene C (**3**)...5
Figure S2. Key NOESY correlations and relative configuration for munropenes C-D (**3**-**4**) (protons of methyl groups are omitted)...5
Figure S3. Key NOESY correlations and relative configuration for munropenes E-F (**5**-**6**) (protons of methyl groups are omitted)..5
Figure S4. Effects of different concentrations of munropenes A-F (compounds **1**-**6**) on HCT116 cells viability. 8
Figure S5. ^{1}H NMR spectrum of munropene A (**1**) in CD_3OD...9
Figure S6. ^{13}C NMR spectrum of munropene A (**1**) in CD_3OD. 9
Figure S7. ^{1}H–^{1}H COSY spectrum of munropene A (**1**) in CD_3OD................................10
Figure S8. HSQC spectrum of munropene A (**1**) in CD_3OD...10
Figure S9. HMBC spectrum of munropene A (**1**) in CD_3OD...11
Figure S10. ROESY spectrum of munropene A (**1**) in CD_3OD. 11
Figure S11. IR spectrum of munropene A (**1**)..12
Figure S12. ^{1}H NMR spectrum of munropene B (**2**) in CD_3OD......................................13
Figure S13. ^{13}C NMR spectrum of munropene B (**2**) in CD_3OD.....................................13
Figure S14. ^{1}H–^{1}H COSY spectrum of munropene B (**2**) in CD_3OD. 14
Figure S15. HSQC spectrum of munropene B (**2**) in CD_3OD...14
Figure S16. HMBC spectrum of munropene B (**2**) in CD_3OD...15
Figure S17. ROESY spectrum of munropene B (**2**) in CD_3OD...15
Figure S18. HETLOC spectrum of munropene B (**2**) in CD_3OD. 16
Figure S19. PS-HMBC spectrum of munropene B (**2**) in CD_3OD...................................16
Figure S20. PS-COSY spectrum of munropene B (**2**) in CD_3OD. 17
Figure S21. IR spectrum of munropene B (**2**)...17
Figure S22. CD spectrum of munropene B (**2**)..18
Figure S23. ^{1}H NMR spectrum of munropene C (**3**) in CD_3OD. 18
Figure S24. ^{13}C NMR spectrum of munropene C (**3**) in CD_3OD.....................................19
Figure S25. ^{1}H–^{1}H COSY spectrum of munropene C (**3**) in CD_3OD..............................19
Figure S26. HSQC spectrum of munropene C (**3**) in CD_3OD..20
Figure S27. HMBC spectrum of munropene C (**3**) in CD_3OD. 20
Figure S28. ROESY spectrum of munropene C (**3**) in CD_3OD...21
Figure S29. HETLOC spectrum of munropene C (**3**) in CD_3OD...................................21
Figure S30. PS-HMBC spectrum of munropene C (**3**) in CD_3OD. 22
Figure S31. PS-COSY spectrum of munropene C (**3**) in CD_3OD...................................22
Figure S32. IR spectrum of munropene C (**3**). 23
Figure S32. CD spectrum of munropene C (**3**). 23
Figure S34. ^{1}H NMR spectrum of munropene D (**4**) in CD_3OD. 24

Figure S35. ^{13}C NMR spectrum of munropene D (**4**) in CD_3OD. 24
Figure S36. ^1H–^1H COSY spectrum of munropene D (**4**) in CD_3OD..............................25
Figure S37. HSQC spectrum of munropene D (**4**) in CD_3OD..25
Figure S38. HMBC spectrum of munropene D (**4**) in CD_3OD..26
Figure S39. ROESY spectrum of munropene D (**4**) in CD_3OD. 26
Figure S40. HETLOC spectrum of munropene D (**4**) in CD_3OD.....................................27
Figure S41. PS-HMBC spectrum of munropene D (**4**) in CD_3OD. 27
Figure S42. PS-COSY spectrum of munropene D (**4**) in CD_3OD....................................28
Figure S43. IR spectrum of munropene D (**4**)..28
Figure S44. CD spectrum of munropene D (**4**). 29
Figure S45. ^1H NMR spectrum of munropene E (**5**) in CD_3OD.......................................30
Figure S46. ^{13}C NMR spectrum of munropene E (**5**) in CD_3OD. 30
Figure S47. ^1H–^1H COSY spectrum of munropene E (**5**) in CD_3OD. 31
Figure S48. HSQC spectrum of munropene E (**5**) in CD_3OD..31
Figure S49. HMBC spectrum of munropene E (**5**) in CD_3OD..32
Figure S50. ROESY spectrum of munropene E (**5**) in CD_3OD. 32
Figure S51. HETLOC spectrum of munropene E (**5**) in CD_3OD. 33
Figure S52. PS-HMBC spectrum of munropene E (**5**) in CD_3OD...................................33
Figure S53. IR spectrum of munropene E (**5**)...34
Figure S54. ^1H NMR spectrum of munropene F (**6**) in CD_3OD. 34
Figure S55. ^{13}C NMR spectrum of munropene F (**6**) in CD_3OD. 35
Figure S56. ^1H–^1H COSY spectrum of munropene F (**6**) in CD_3OD. 35
Figure S57. HSQC spectrum of munropene F (**6**) in CD_3OD..36
Figure S58. HMBC spectrum of munropene F (**6**) in CD_3OD..36
Figure S59. ROESY spectrum of munropene F (**6**) in CD_3OD. 37
Figure S60. HETLOC spectrum of munropene F (**6**) in CD_3OD.....................................37
Figure S61. PS-HMBC spectrum of munropene F (**6**) in CD_3OD...................................38
Figure S62. IR spectrum of munropene F (**6**)...38

Table S1. Partial $^3J_{H,H}$ and $^{2,3}J_{C,H}$ data for munropenes A-F (**1**-**6**).

Position	**1**	**2**	**3**	**4**	**5**	**6**
^3J (H-24,H-23)	8.6	8.5	9.5	7.8	8.7	6.9
^3J (H-24,C-22)	2.2	3.1	3.3	-1.5	1.7	2.3
^3J (C-25,H-23)	6.5	5.9	4.9	2.8	4.7	3.7
^3J (H-24,C-23)	-3.2	-3.4	-3.2	3.2	-3.2	-3.2
^3J (H-23, H-22a)	8.2	12.0	8.6	8.8	9.1	6.7
^3J (H-23, H-22b)	6.5	4.0	5.4	2.8	9.0	6.7
^3J (H-23, C-20)	5.5	4.0	2.5	2.6	4.5	2.8
^3J (C-24, H-22a)	2.1	-1.6	2.0	0.6	-1.9	Nd
^3J (C-24, H-22b)	1.9	-1.7	2.0	1.0	1.6	1.2
^2J (C-23, H-22a)	-6.0	6.8	-6.8	-5.5	4.8	5.3
^2J (C-23, H-22b)	-1.7	3.3	-2.0	-3.9	-2.5	-3.7
^3J (H-20,H-22a)	5.8	8.0	11.0	6.7	4.7	3.4
^3J (H-20,H-22b)	6.1	5.0	4.9	4.9	4.1	4.3
^3J (H-20,C-23)	3.1	5.8	3.1	2.7	Nd	Nd
^3J (C-17, H-22a)	-2.3	3.5	2.1	2.5	6.5	2.4
^3J (C-17, H-22b)	1.6	3.5	-3.3	-4.3	3.5	-1.7
^2J (C-20, H-22a)	2.6	5.0	5.1	5.6	6.5	5.4
^2J (C-20, H-22b)	2.3	3.5	-1.7	-4.0	3.4	3.5
^3J (H-17,H-20)	16.0	8.1	13.7	12.2	8.8	9.8
^3J (H-17,C-21)	3.7	4.2	-5.9	1.6	2.8	3.9
^3J (H-17,C-22)	3.8	5.2	2.9	3.5	2.6	2.2
^3J (C-16,H-20)	Nd	Nd	Nd	Nd	Nd	Nd
^3J (C-13,H-20)	2.8	2.9	2.6	3.6	2.9	2.9
^3J (H-1,C-5)		7.3	4.2			
^3J (H-1,C-9)		6.1	6.2			
^3J (H-1,C-19)		1.2	4.1			
^3J (H-1,H-2a)		7.8	11.2			
^3J (H-1,H-2b)		4.0	6.4			
^3J (H-1,C-3)		6.2	4.6			
^2J (C-10, H-2a)		9.0	4.8			
^2J (C-10, H-2b)		Nd	Nd			

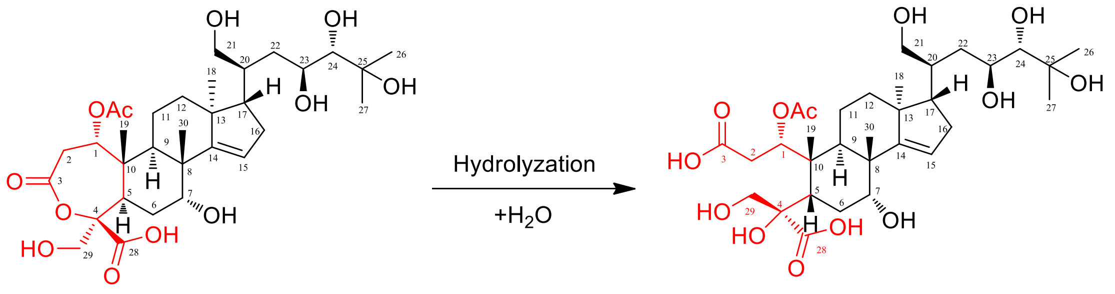

1 **3**

Figure S1. Possible biogenetic pathway of munropene C (**3**).

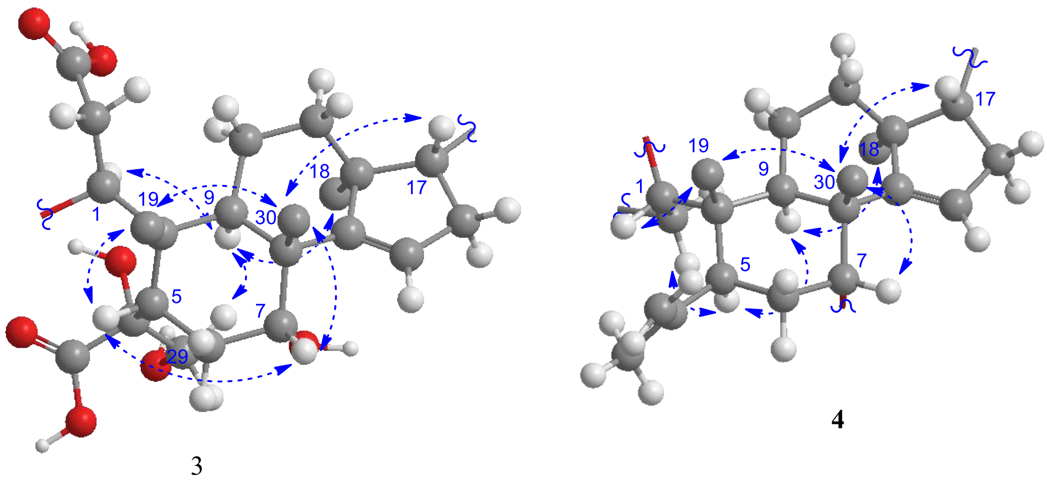

3 **4**

Figure S2. Key NOESY correlations and relative configuration for munropenes C-D (**3**-**4**) (protons of methyl groups are omitted).

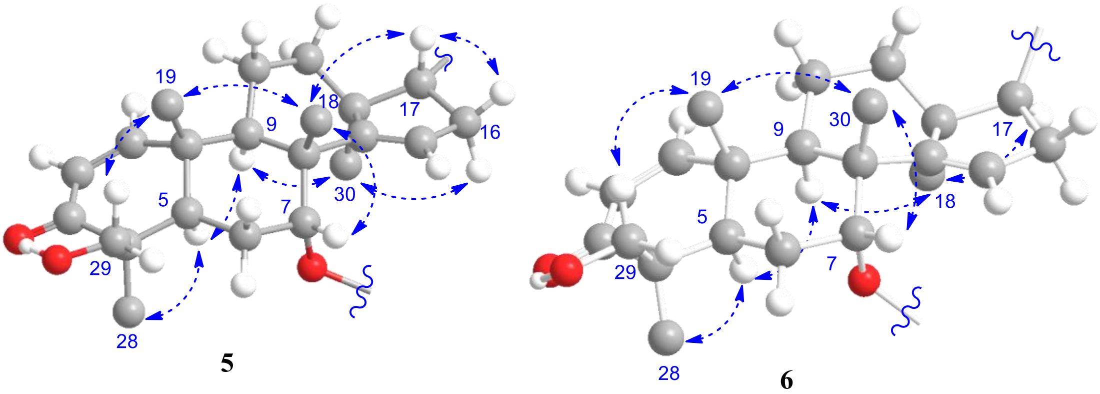

5 **6**

Figure S3. Key NOESY correlations and relative configuration for munropenes E-F (**5**-**6**) (protons of methyl groups are omitted).

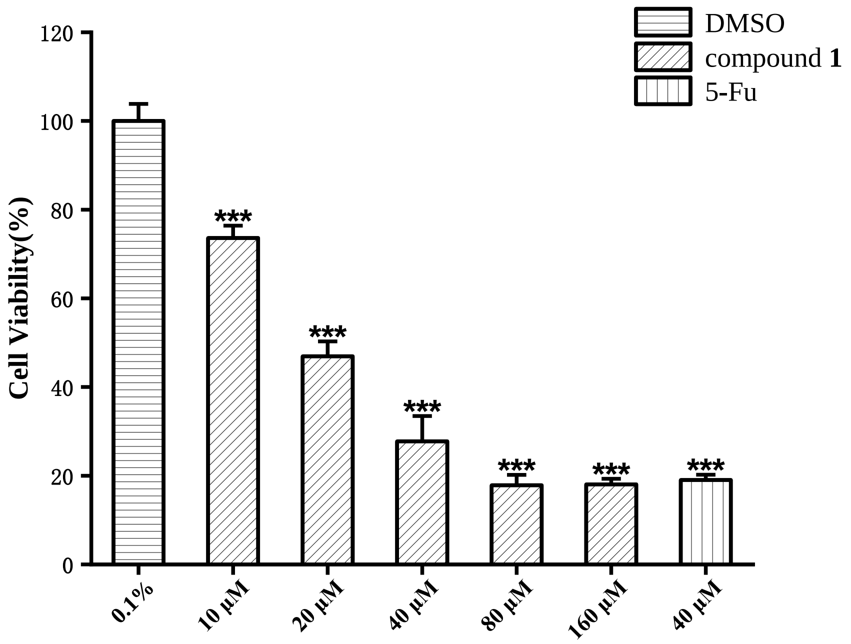
DMSO
compound 1
5-Fu
Cell Viability(%)
0
20
40
60
80
100
120
***
0.1%
10 μM
20 μM
40 μM
80 μM
160 μM
40 μM

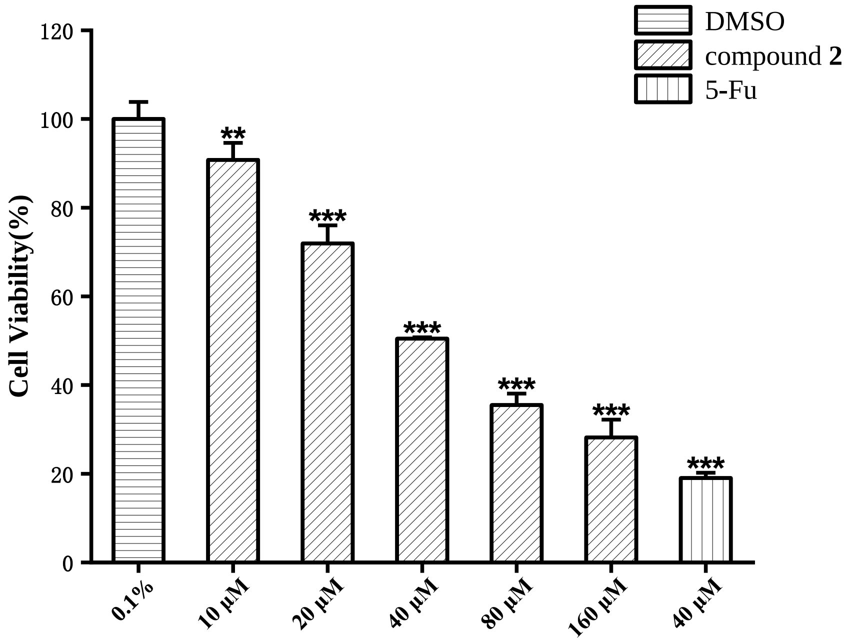
DMSO
compound 2
5-Fu
Cell Viability(%)
0
20
40
60
80
100
120
**
***
0.1%
10 μM
20 μM
40 μM
80 μM
160 μM
40 μM

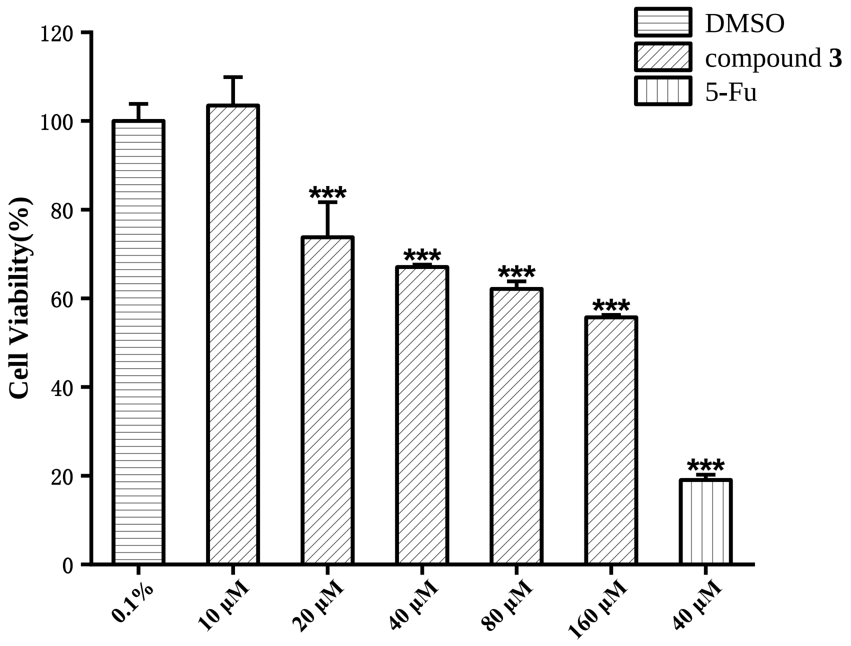

DMSO
compound 3
5-Fu
Cell Viability(%)
0
20
40
60
80
100
120
***
0.1%
10 μM
20 μM
40 μM
80 μM
160 μM
40 μM

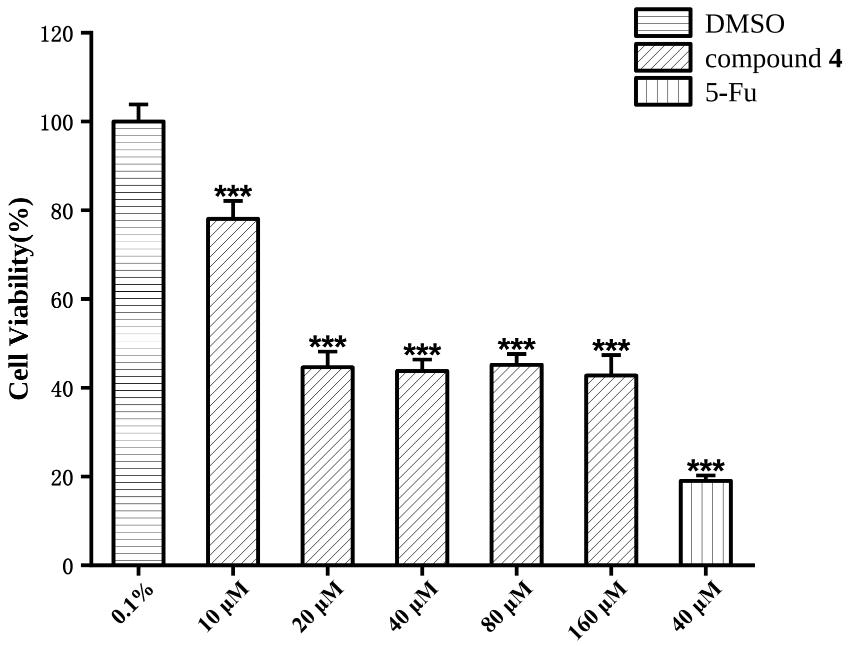

DMSO
compound 4
5-Fu
Cell Viability(%)
0
20
40
60
80
100
120
***
0.1%
10 μM
20 μM
40 μM
80 μM
160 μM
40 μM

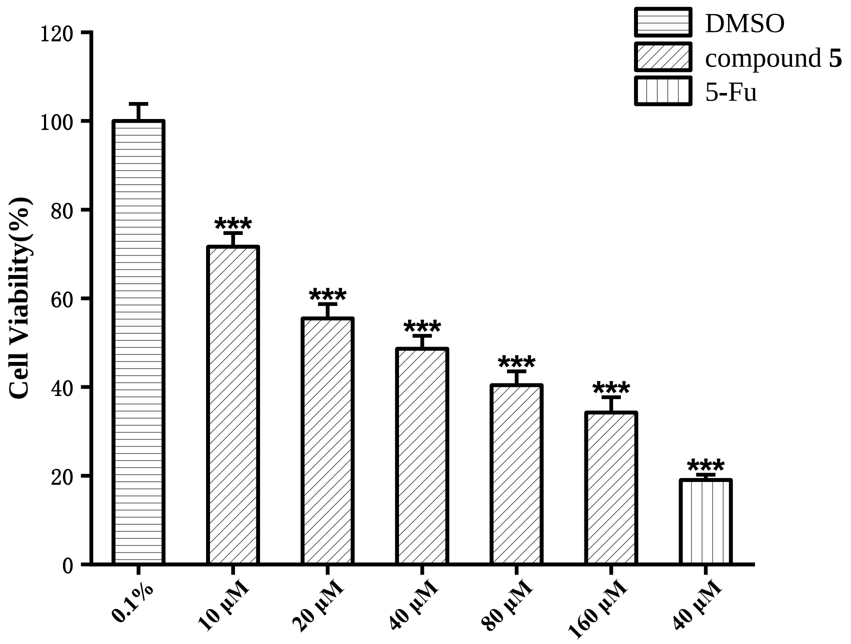

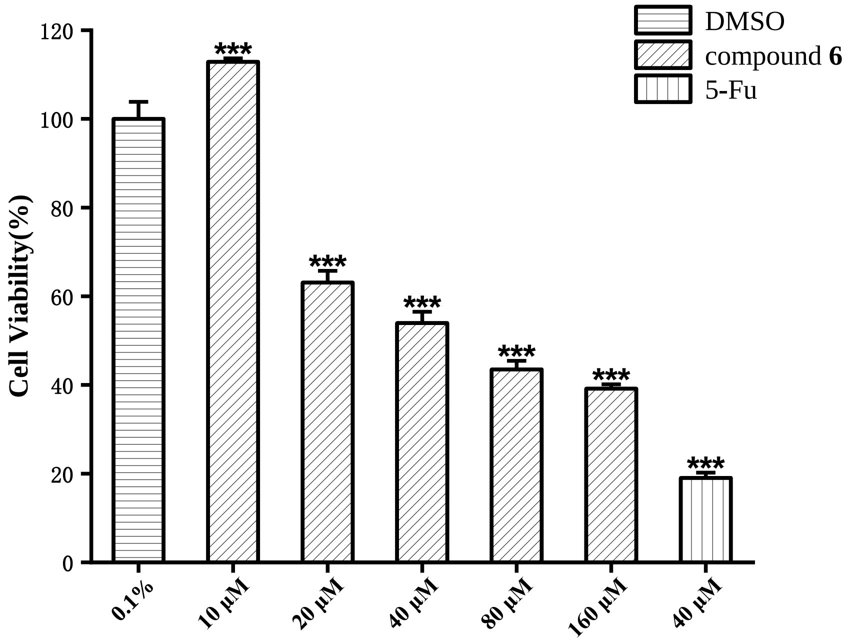

Figure S4. Effects of different concentrations of munropenes A-F (compounds **1**-**6**) proliferation in HCT116 cells at 72 h. Compounds **1**-**6** were tested at concentration ranges from 10 to 160 μM, respectively, and 5-Fu (40 μM) was used as a positive control. Data are represented as the mean ± SD (n = 3). **$p < 0.01$, ***$p < 0.001$ compared to control cells that treated by DMSO. ($p < 0.01$).

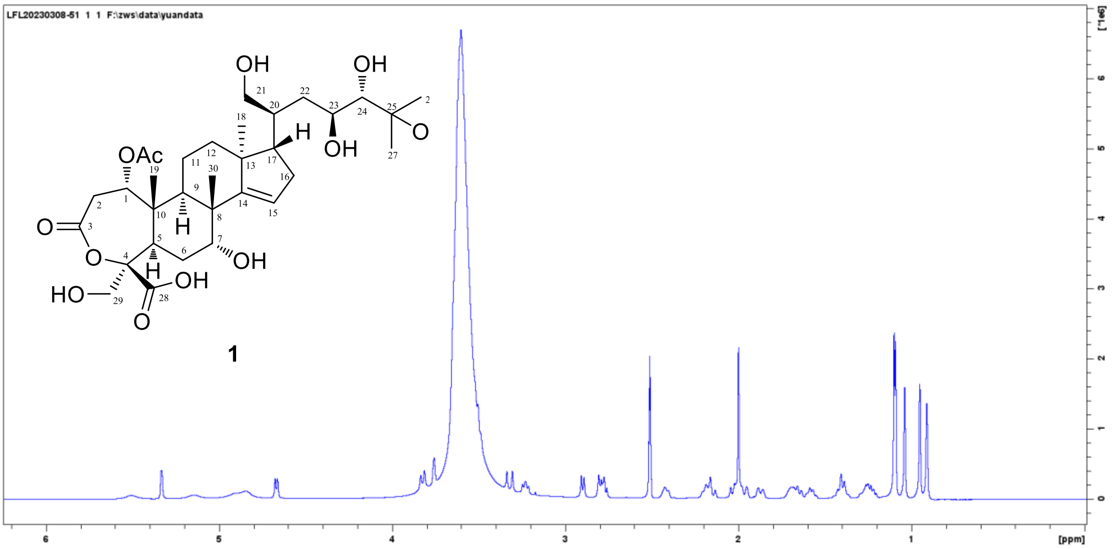

Figure S5. ^{1}H NMR spectrum of munropene A (**1**) in CD_3OD.

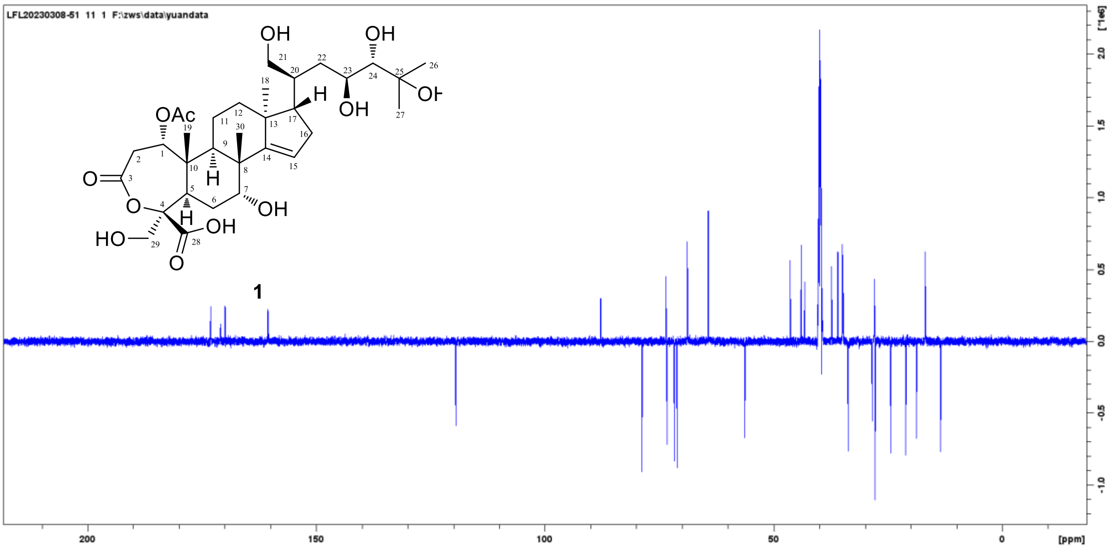

Figure S6. ^{13}C NMR spectrum of munropene A (**1**) in CD_3OD.

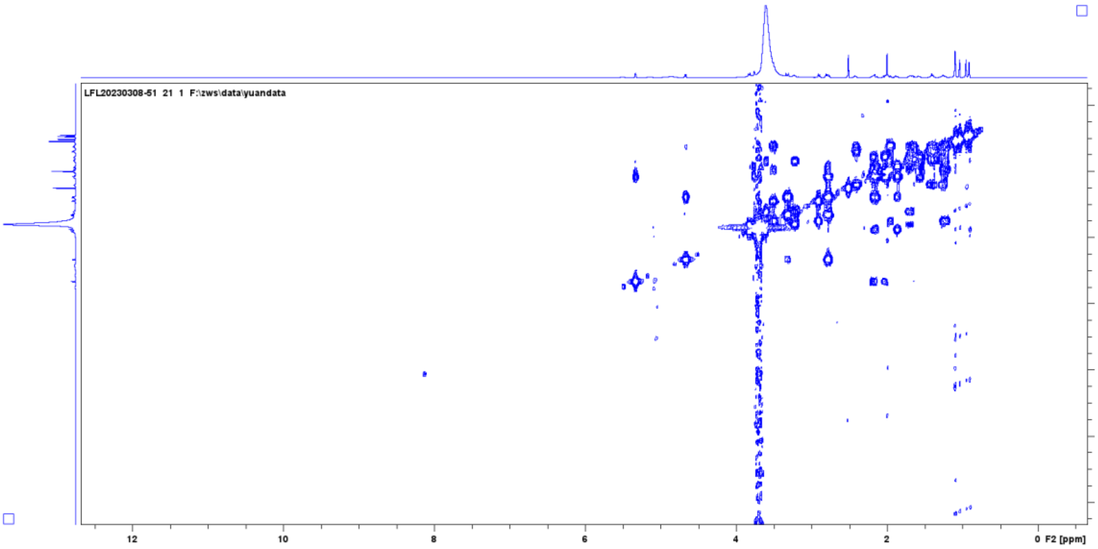

Figure S7. ^{1}H–^{1}H COSY spectrum of munropene A (**1**) in CD_3OD.

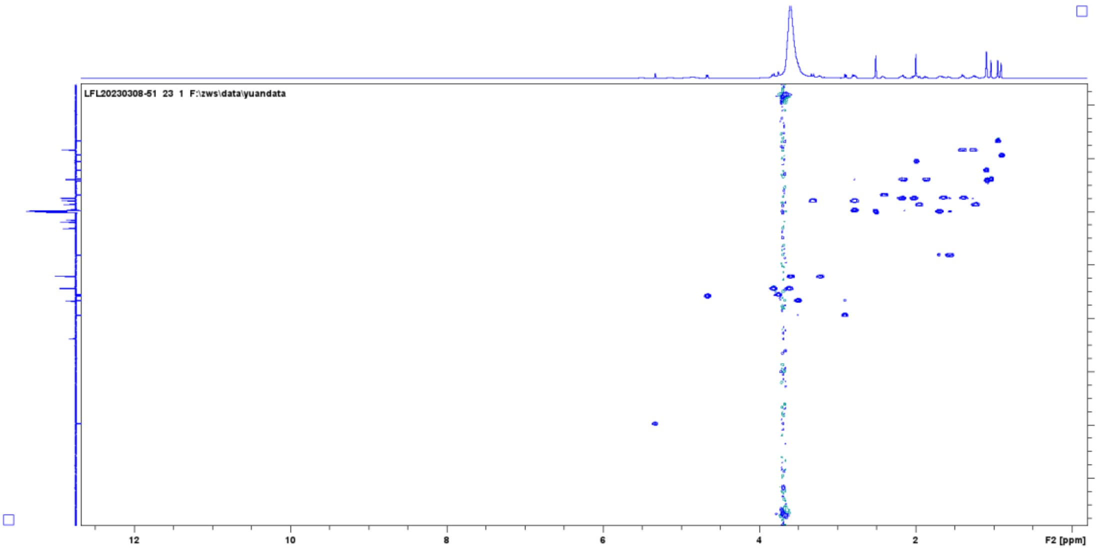

Figure S8. HSQC spectrum of munropene A (**1**) in CD_3OD.

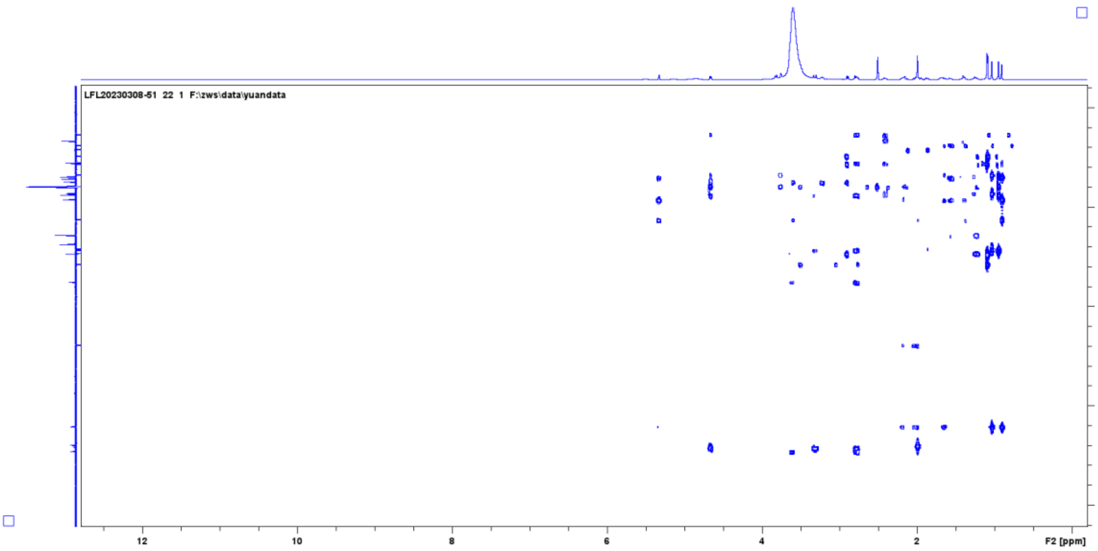

Figure S9. HMBC spectrum of munropene A (**1**) in CD_3OD.

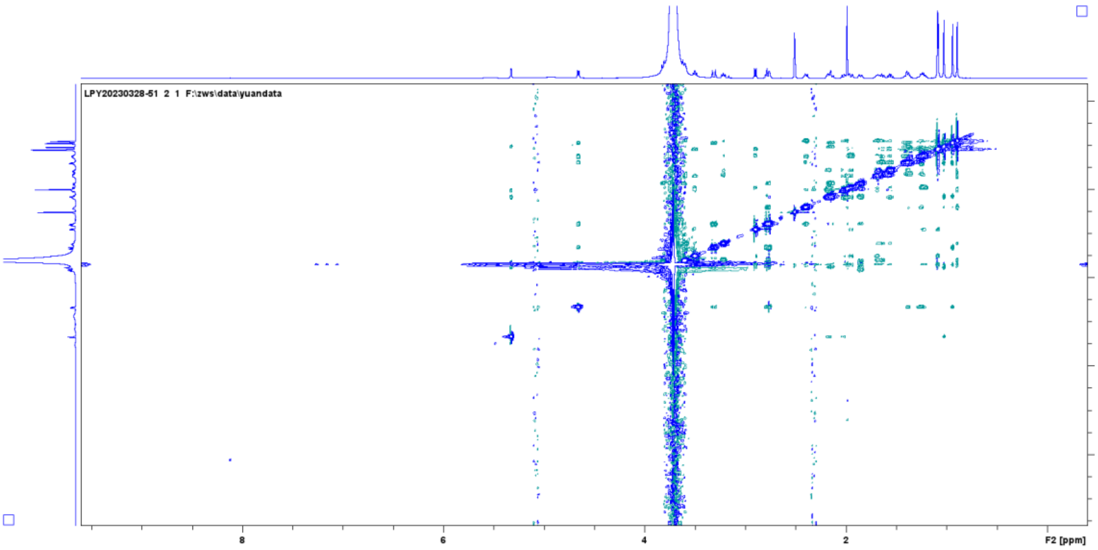

Figure S10. ROESY spectrum of munropene A (**1**) in CD_3OD.

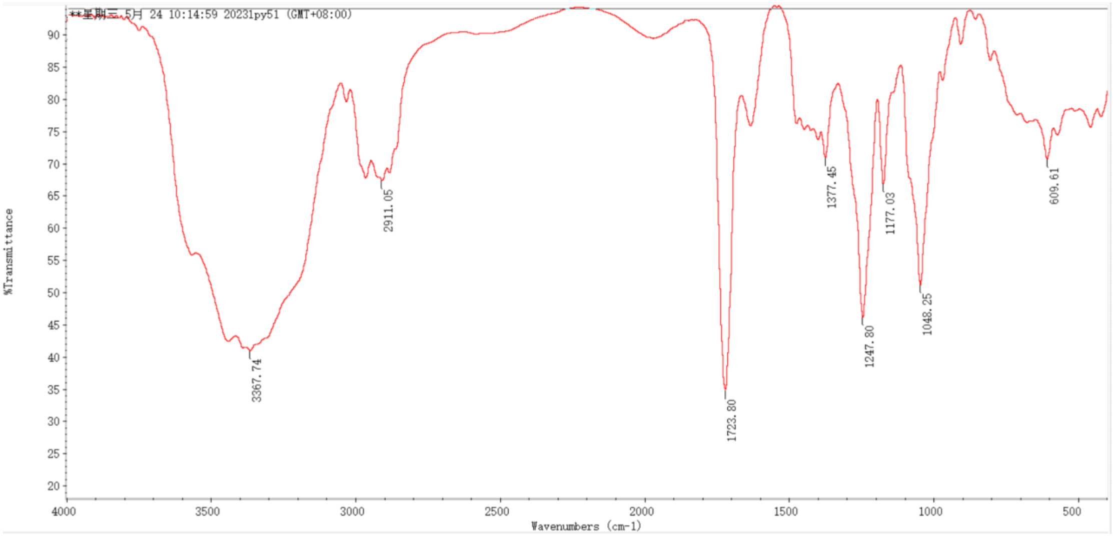

Figure S11. IR spectrum of munropene A (**1**).

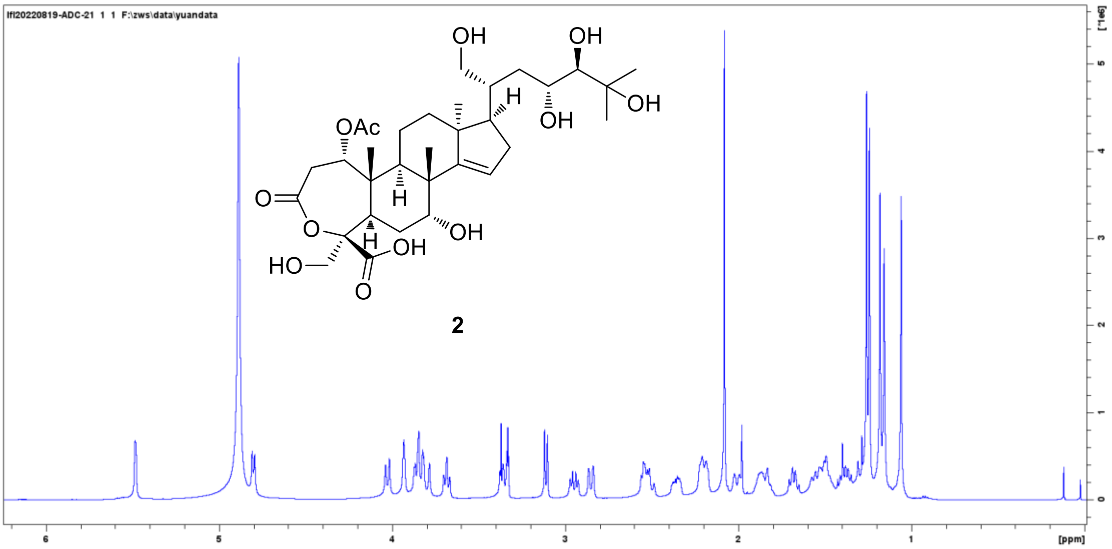

Figure S12. ^{1}H NMR spectrum of munropene B (**2**) in CD3OD.

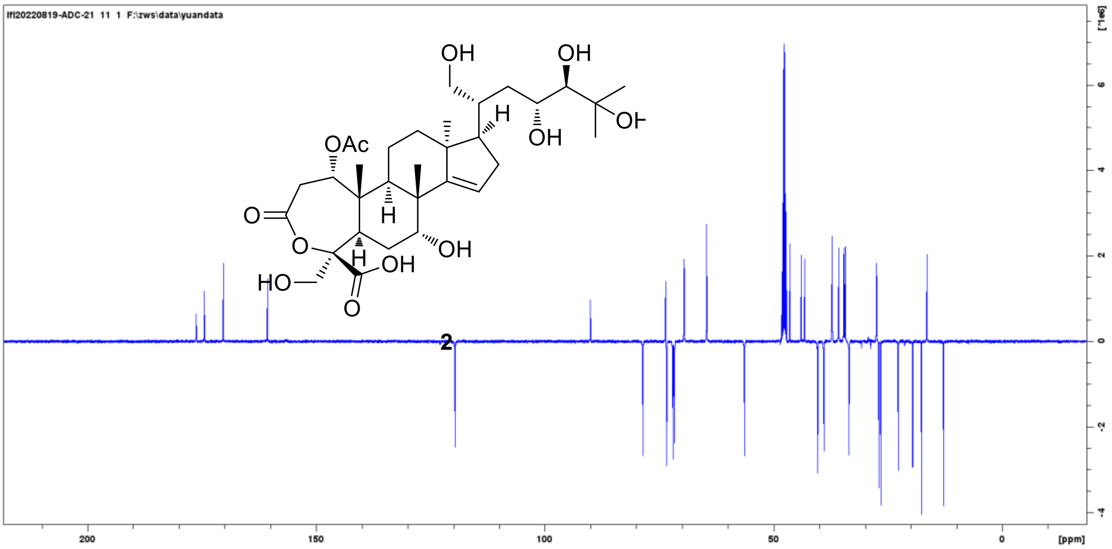

Figure S13. ^{13}C NMR spectrum of munropene B (**2**) in CD3OD.

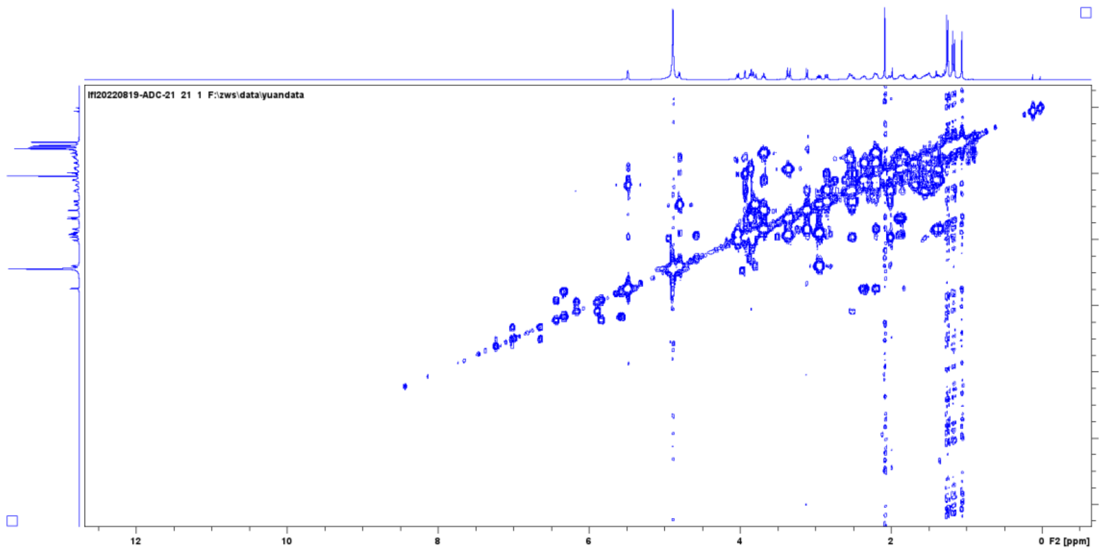

Figure S14. ^{1}H–^{1}H COSY spectrum of munropene B (**2**) in CD_3OD.

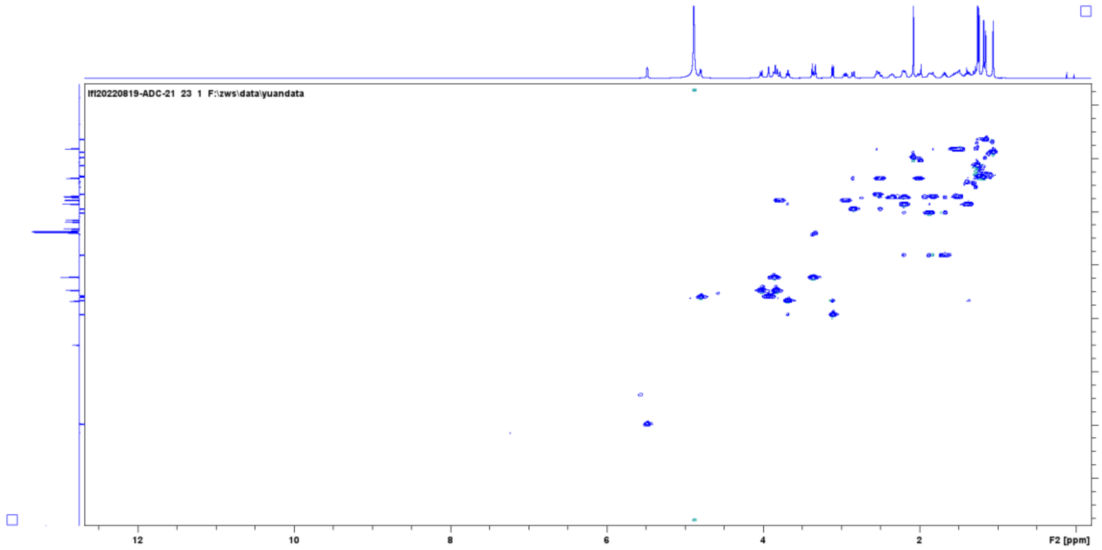

Figure S15. HSQC spectrum of munropene B (**2**) in CD_3OD.

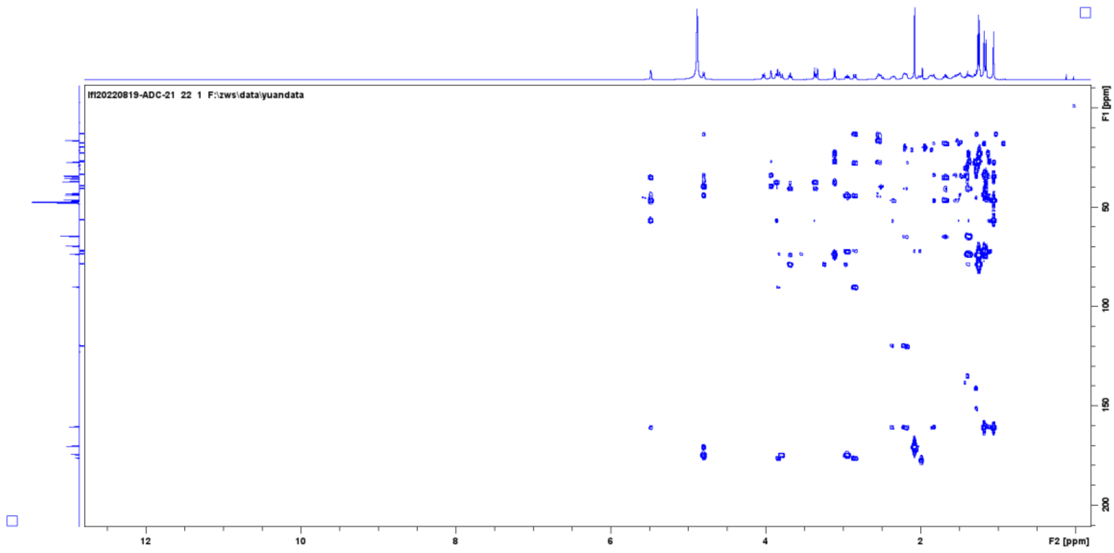

Figure S16. HMBC spectrum of munropene B (**2**) in CD_3OD.

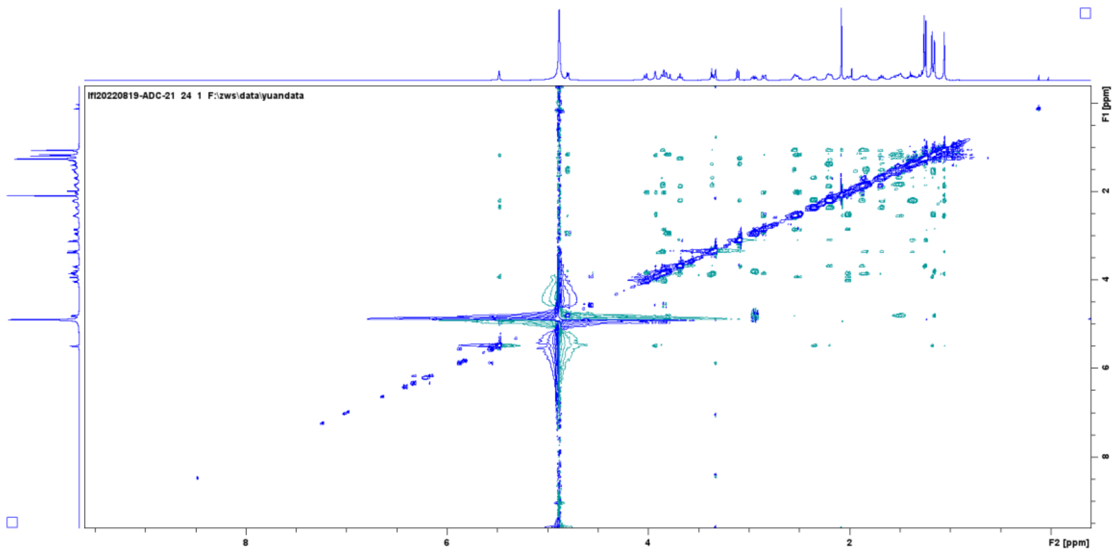

Figure S17. ROESY spectrum of munropene B (**2**) in CD_3OD.

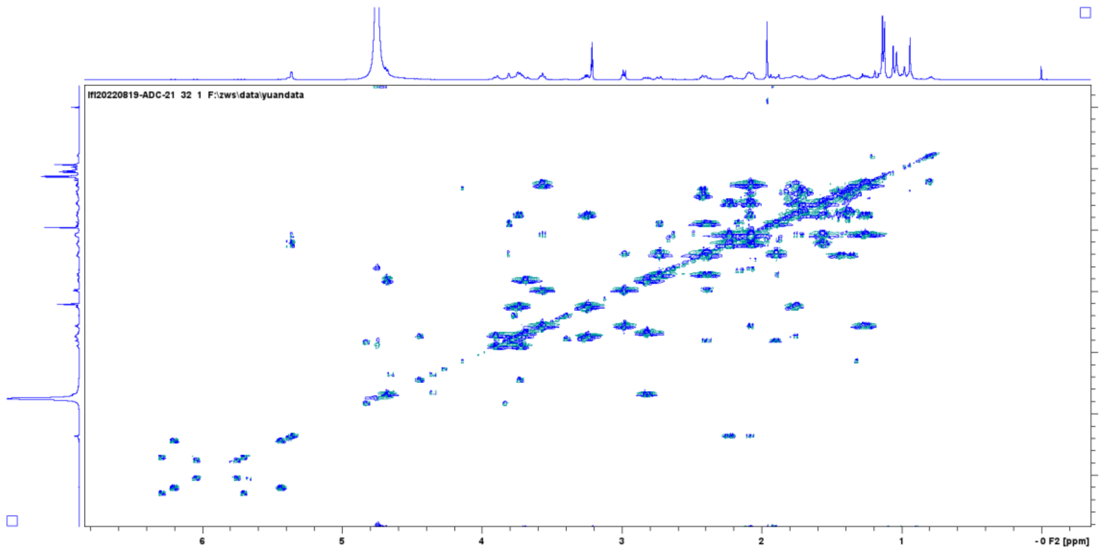

Figure S18. HETLOC spectrum of munropene B (**2**) in CD_3OD.

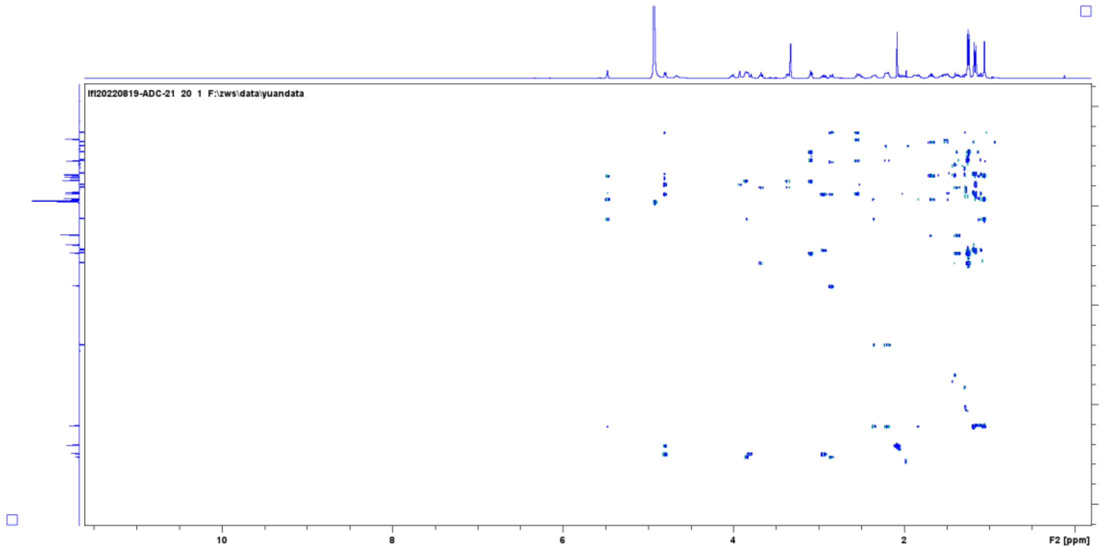

Figure S19. PS-HMBC spectrum of munropene B (**2**) in CD_3OD.

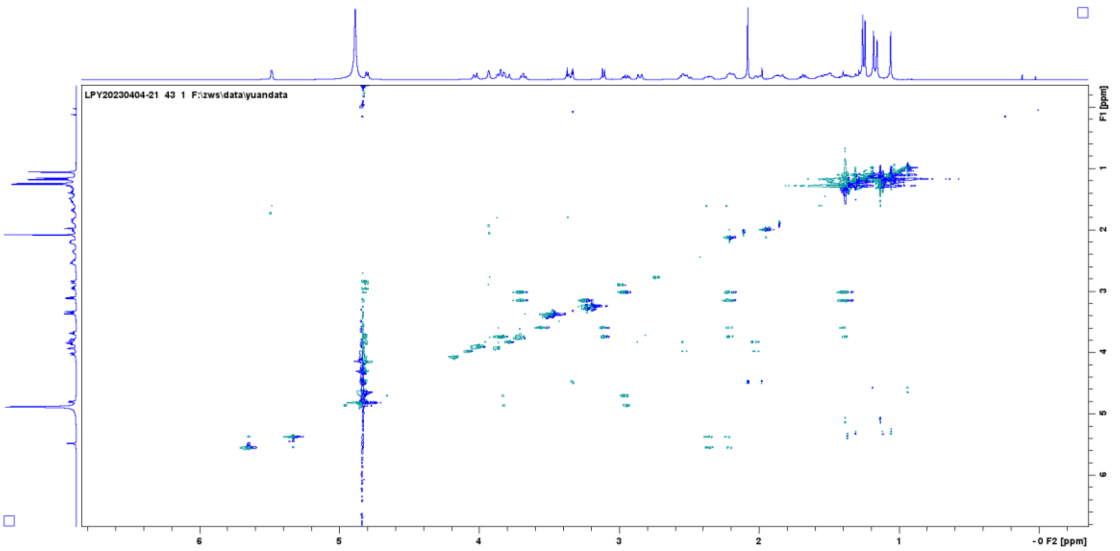

Figure S20. PS-COSY spectrum of munropene B (**2**) in CD_3OD.

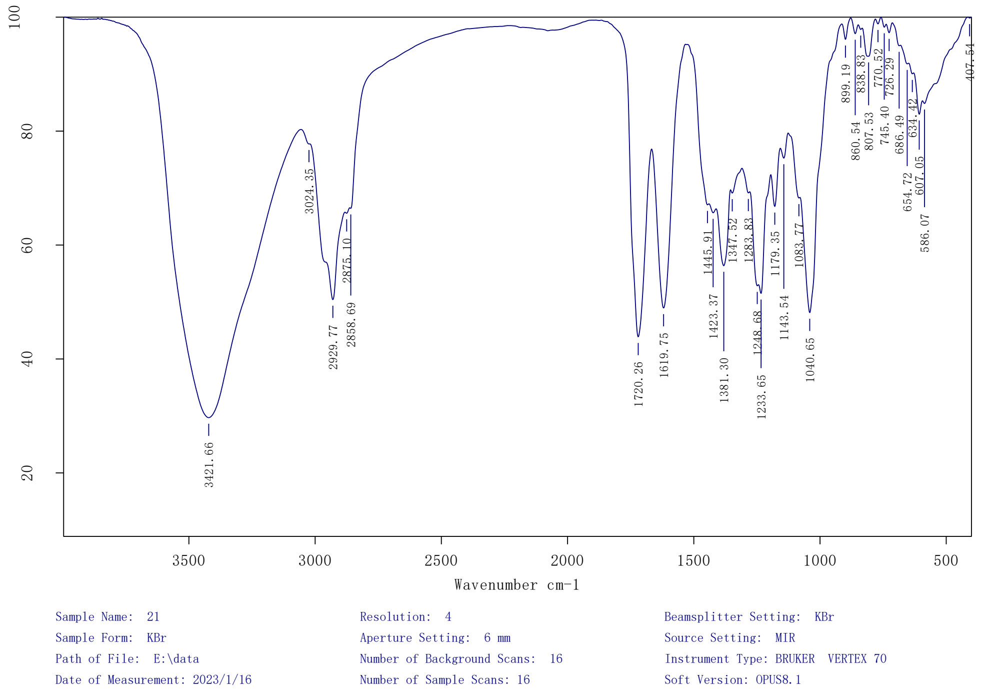

Figure S21. IR spectrum of munropene B (**2**).

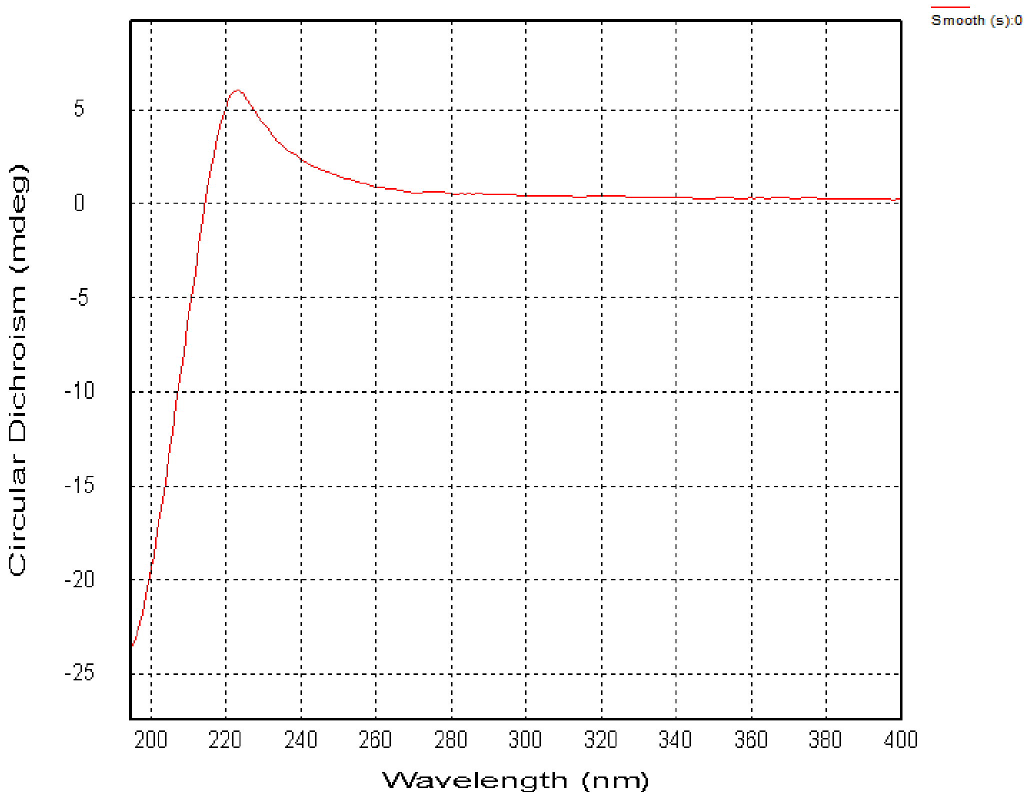

Figure S22. CD spectrum of munropene B (**2**).

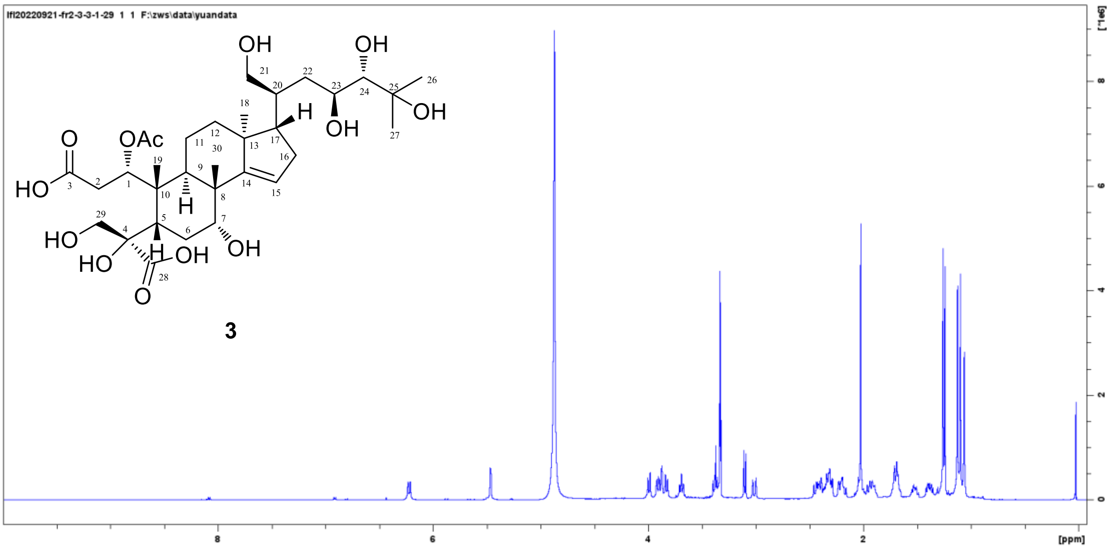

Figure S23. ^{1}H NMR spectrum of munropene C (**3**) in CD_3OD.

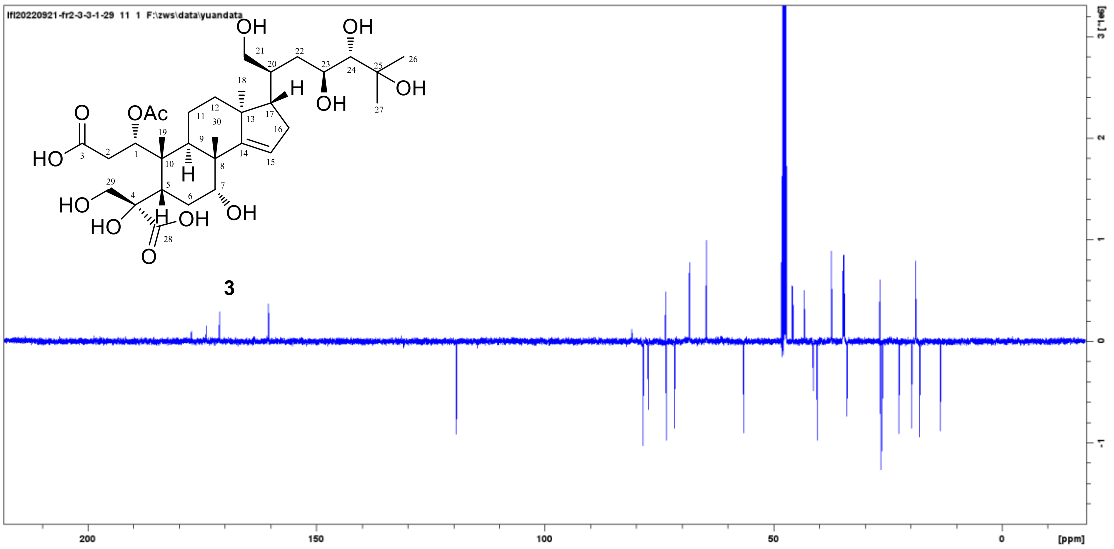

Figure S24. ^{13}C NMR spectrum of munropene C (**3**) in CD3OD.

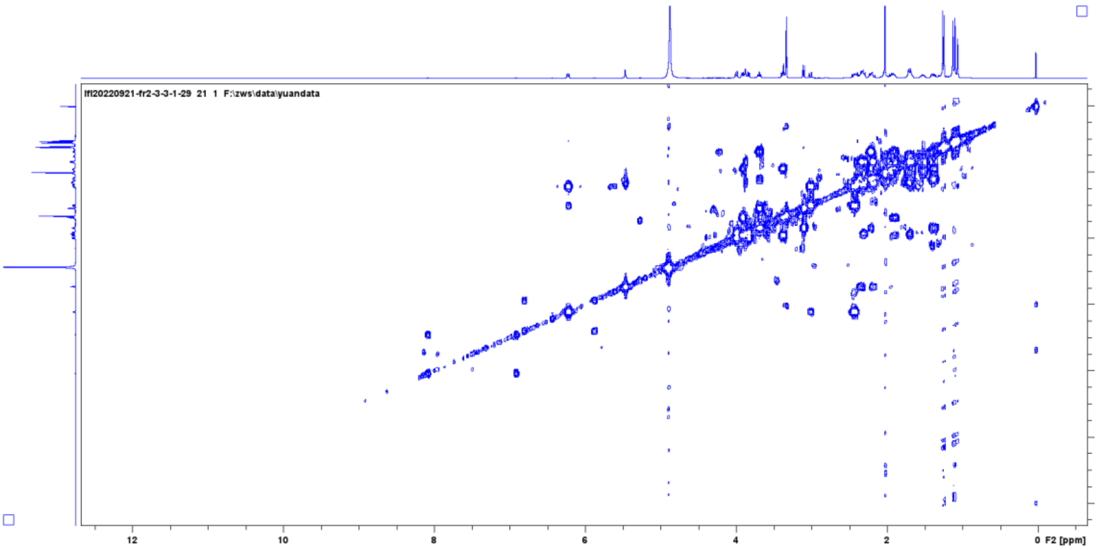

Figure S25. ^{1}H–^{1}H COSY spectrum of munropene C (**3**) in CD3OD..

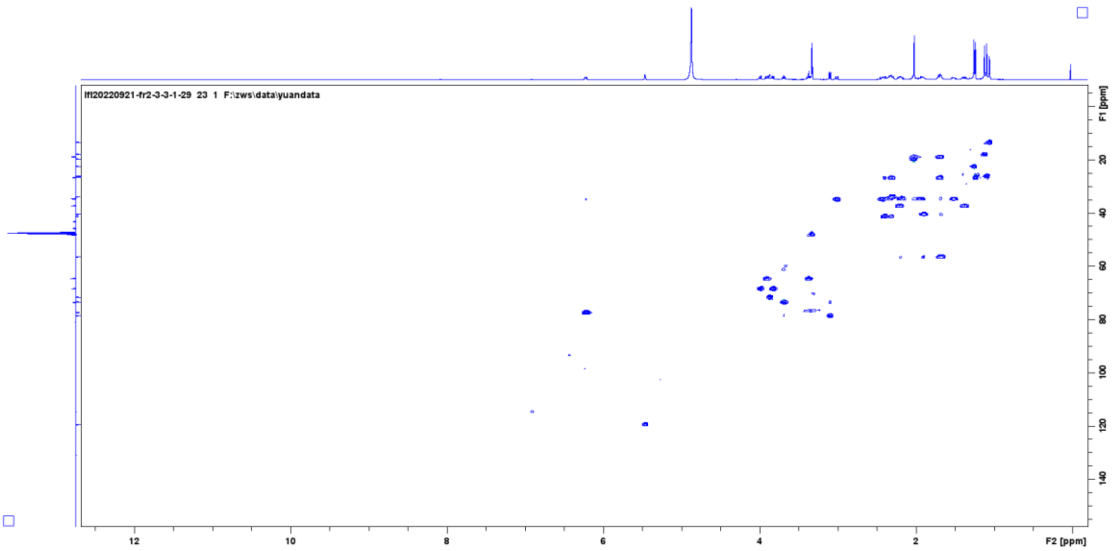

Figure S26. HSQC spectrum of munropene C (**3**) in CD_3OD.

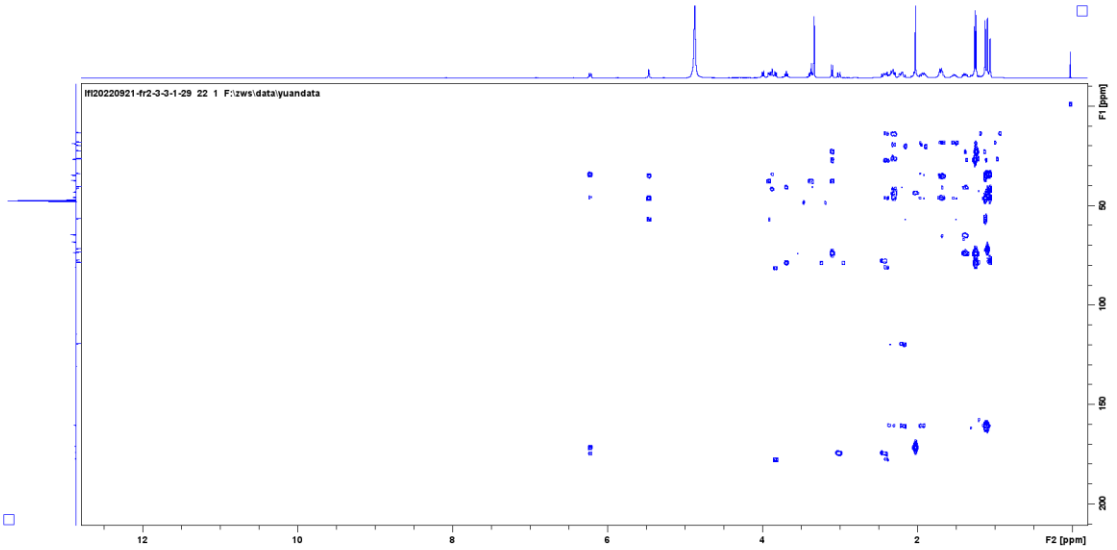

Figure S27. HMBC spectrum of munropene C (**3**) in CD_3OD.

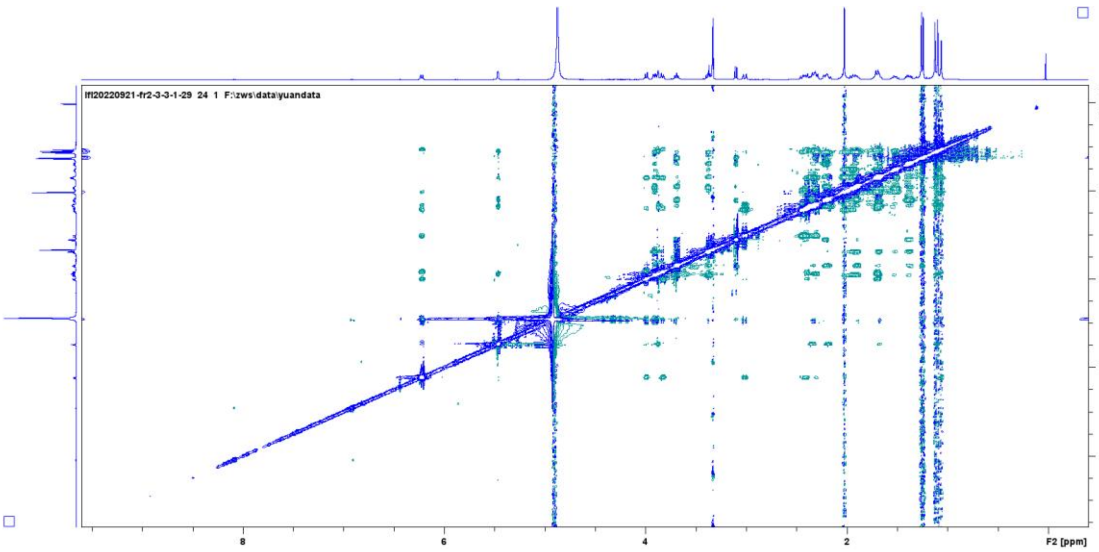

Figure S28. ROESY spectrum of munropene C (**3**) in CD_3OD.

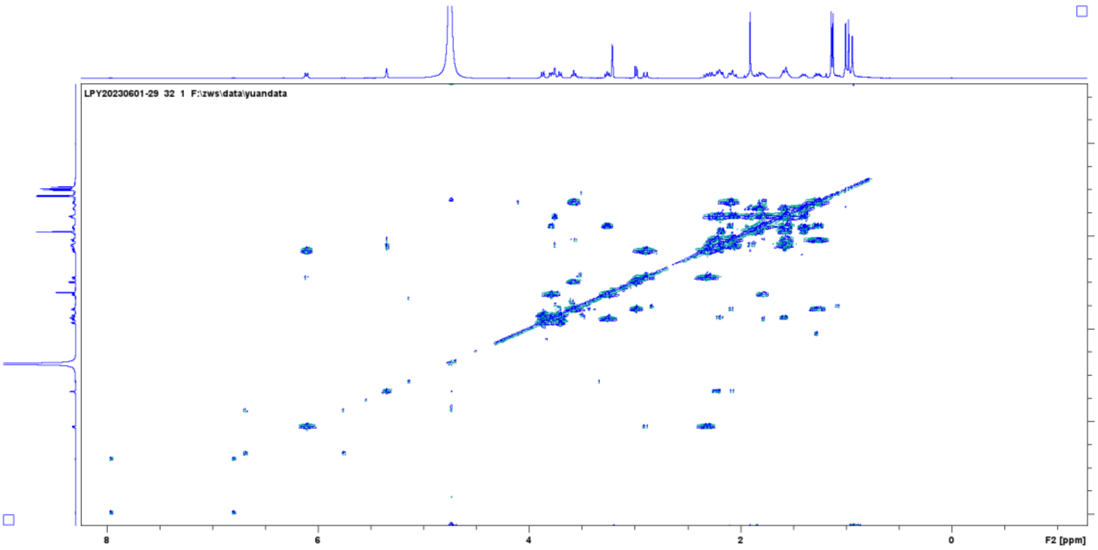

Figure S29. HETLOC spectrum of munropene C (**3**) in CD_3OD.

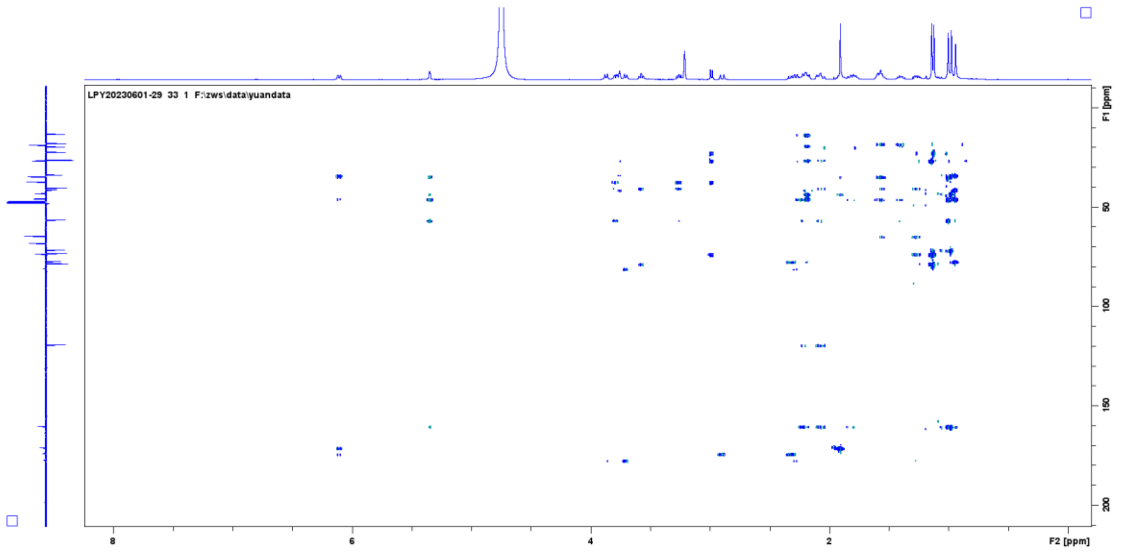

Figure S30. PS-HMBC spectrum of munropene C (**3**) in CD_3OD.

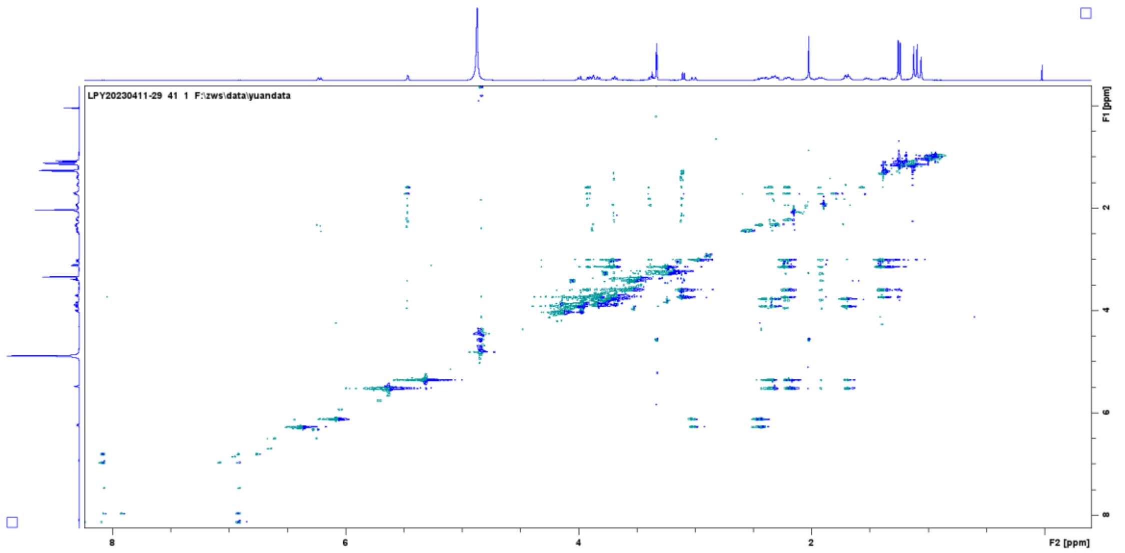

Figure S31. PS-COSY spectrum of munropene C (**3**) in CD_3OD.

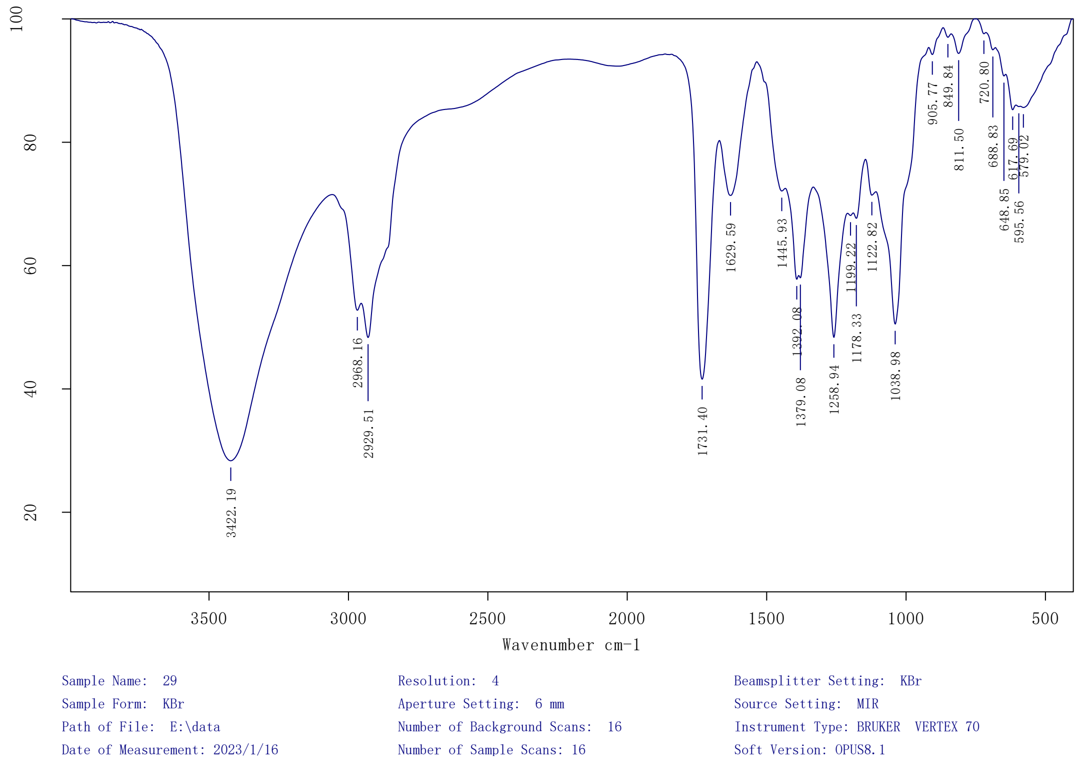

Figure S32. IR spectrum of munropene C (**3**).

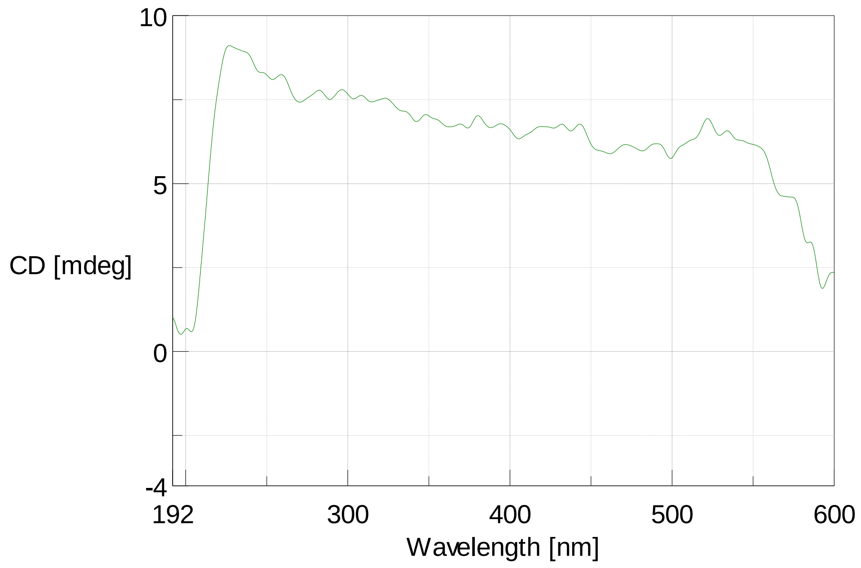

Figure S33. CD spectrum of munropene C (**3**).

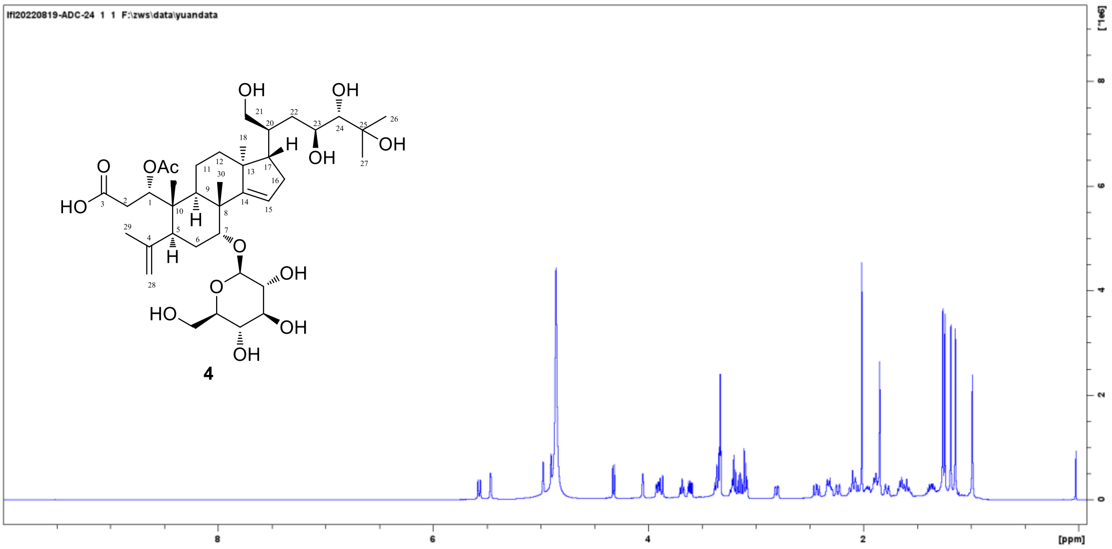

Figure S34. ^{1}H NMR spectrum of munropene D (**4**) in CD_3OD.

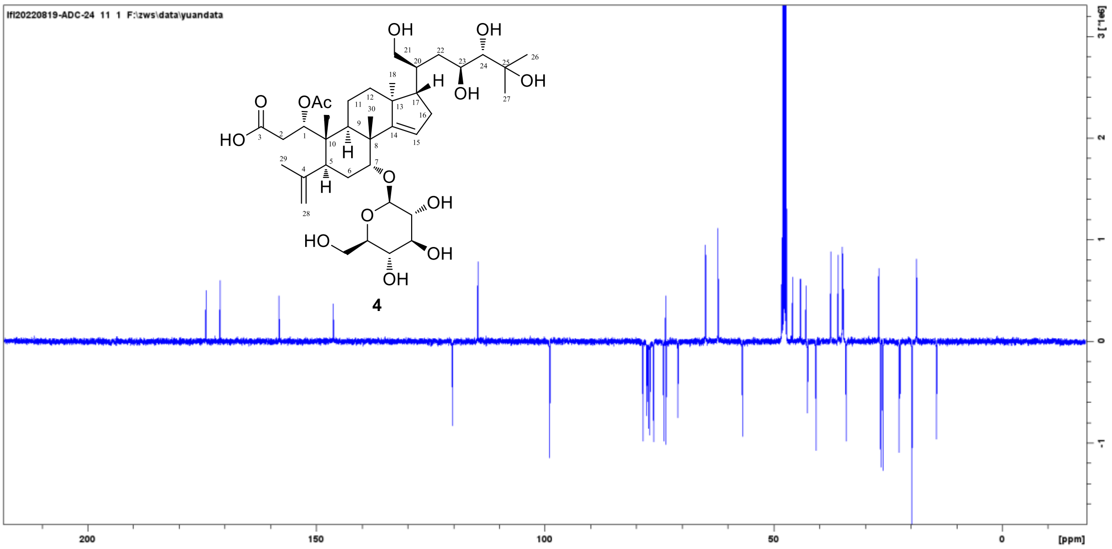

Figure S35. ^{13}C NMR spectrum of munropene D (**4**) in CD_3OD.

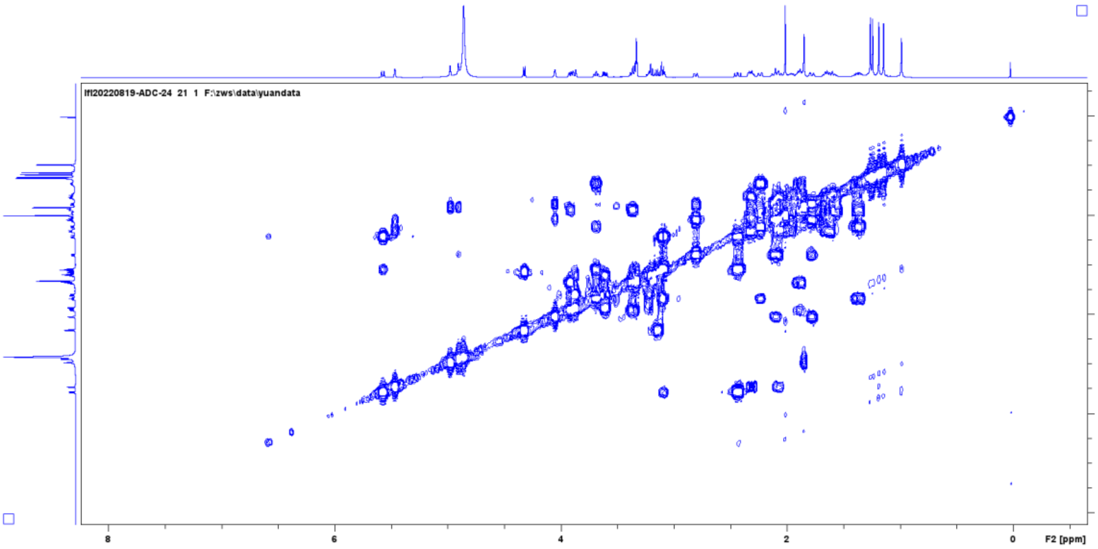

Figure S36. ¹H–¹H COSY spectrum of munropene D (**4**) in CD3OD.

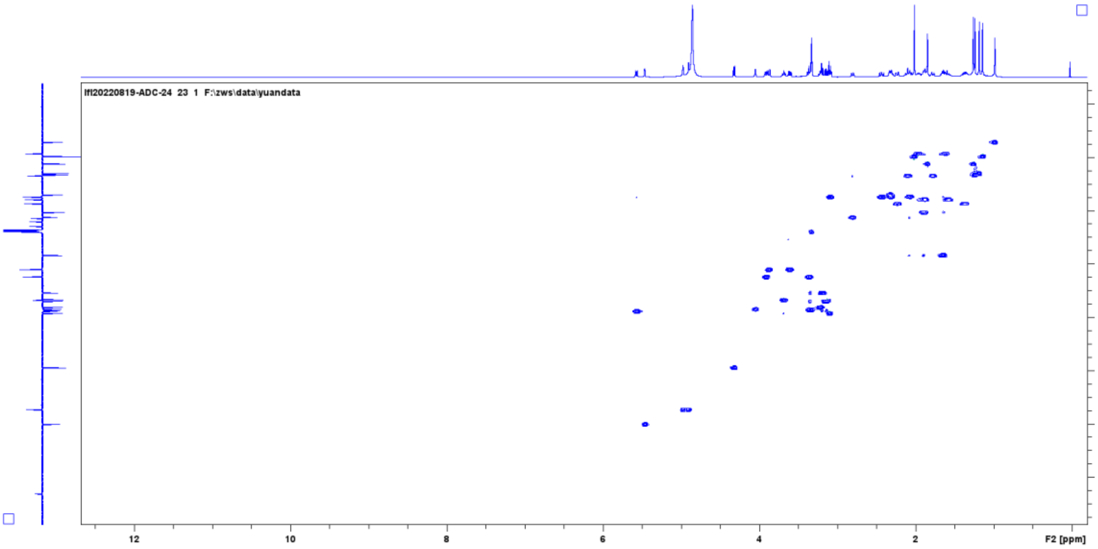

Figure S37. HSQC spectrum of munropene D (**4**) in CD3OD.

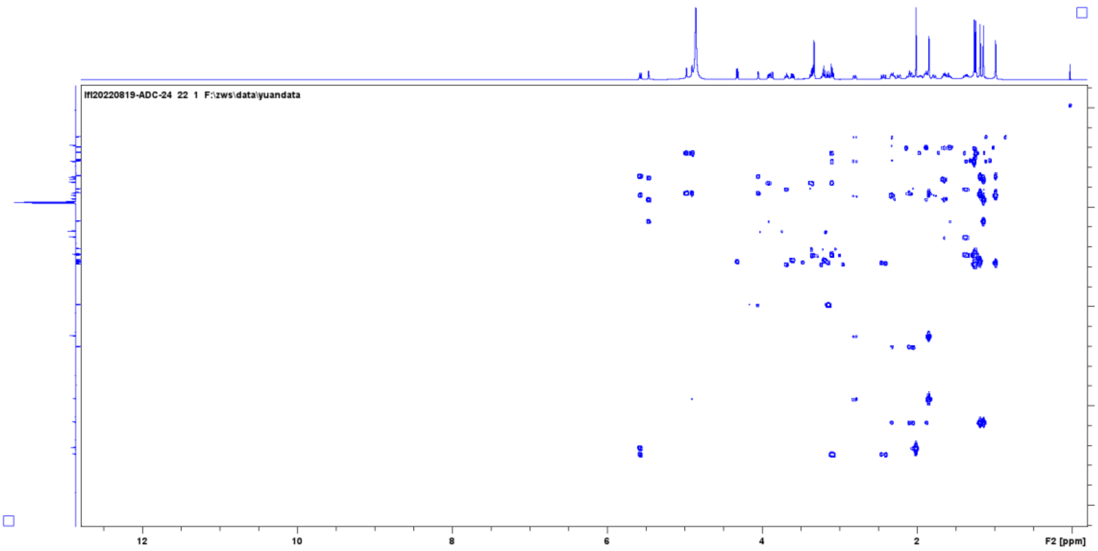

Figure S38. HMBC spectrum of munropene D (**4**) in CD_3OD.

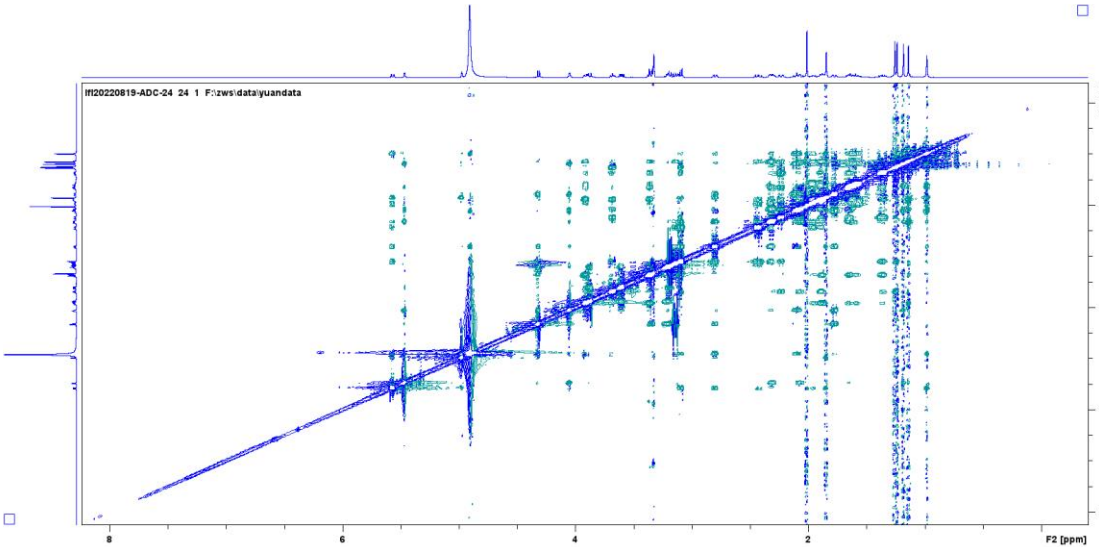

Figure S39. ROESY spectrum of munropene D (**4**) in CD_3OD.

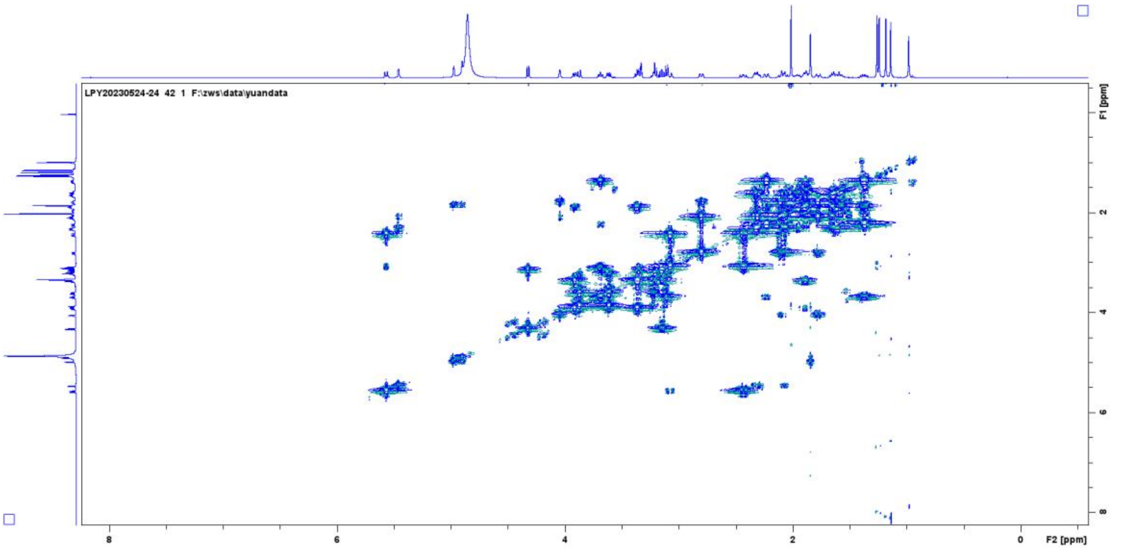

Figure S40. HETLOC spectrum of munropene D (**4**) in CD3OD.

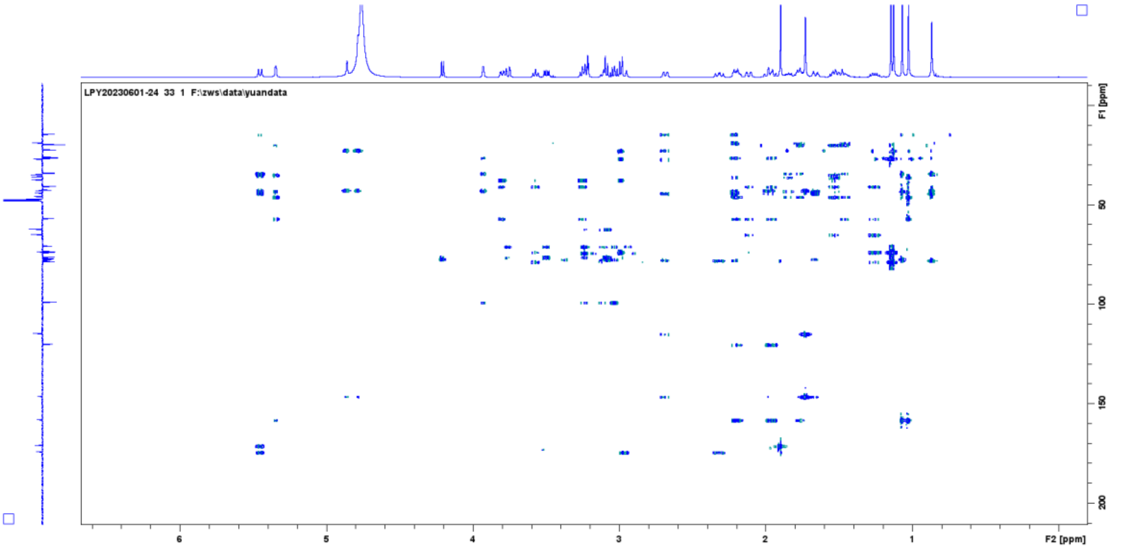

Figure S41. PS-HMBC spectrum of munropene D (4) in CD3OD.

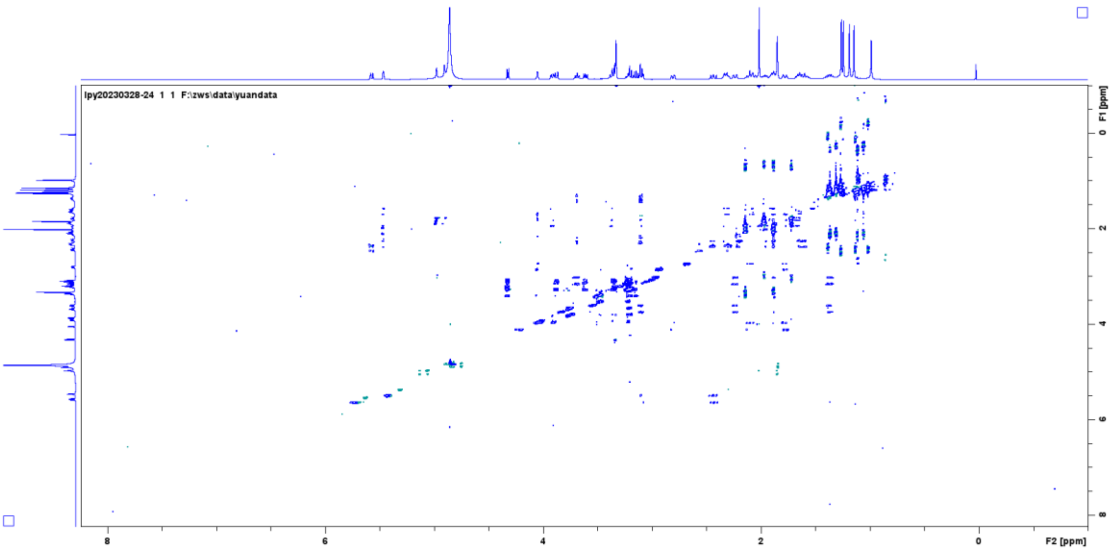

Figure S42. PS-COSY spectrum of munropene D (**4**) in CD_3OD.

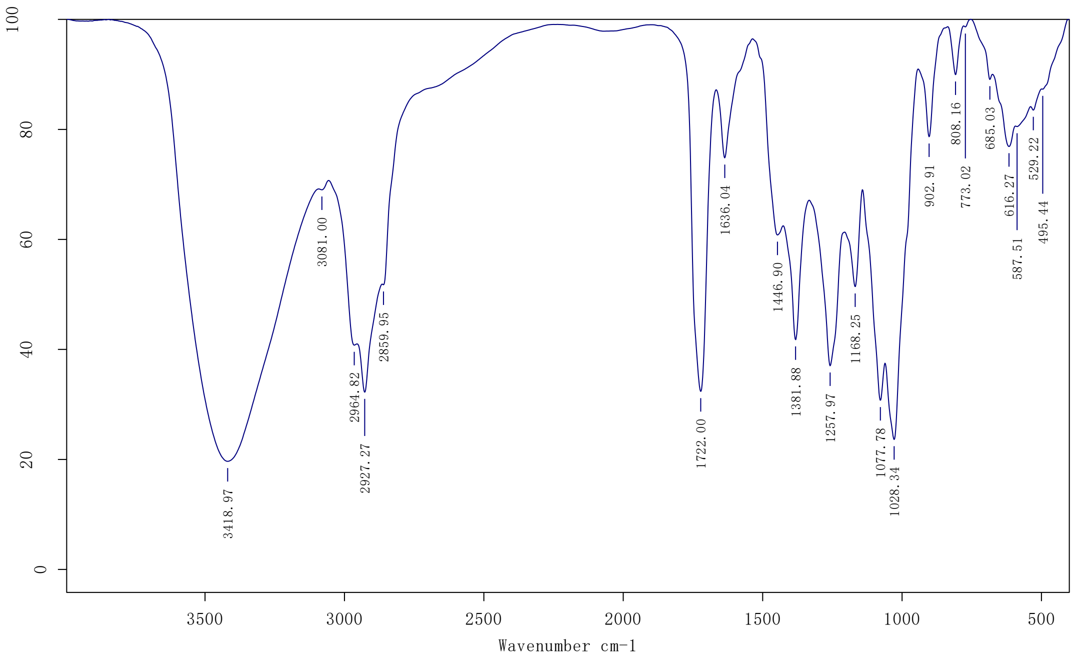

Sample Name: 24
Sample Form: KBr
Path of File: E:\data
Date of Measurement: 2023/1/16
Resolution: 4
Aperture Setting: 6 mm
Number of Background Scans: 16
Number of Sample Scans: 16
Beamsplitter Setting: KBr
Source Setting: MIR
Instrument Type: BRUKER VERTEX 70
Soft Version: OPUS8.1

Figure S43. IR spectrum of munropene D (**4**).

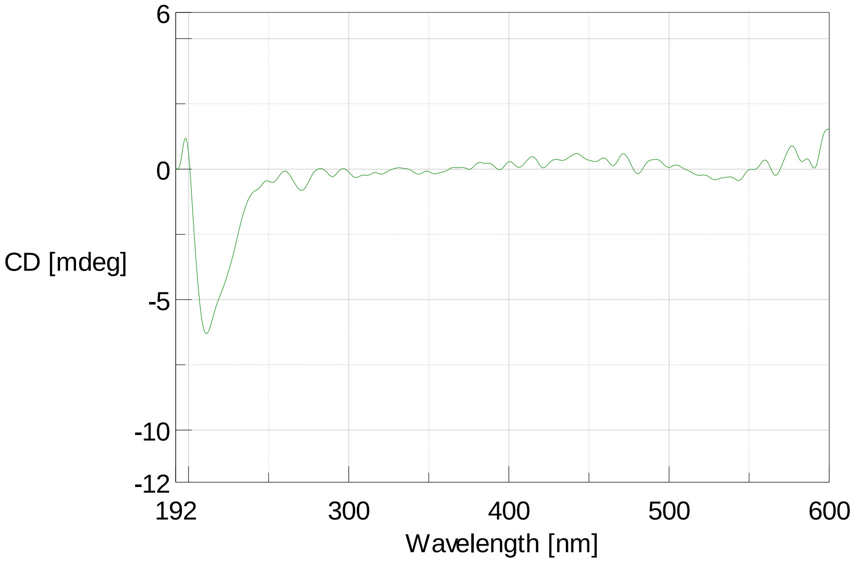

Figure S44. CD spectrum of munropene D (**4**).

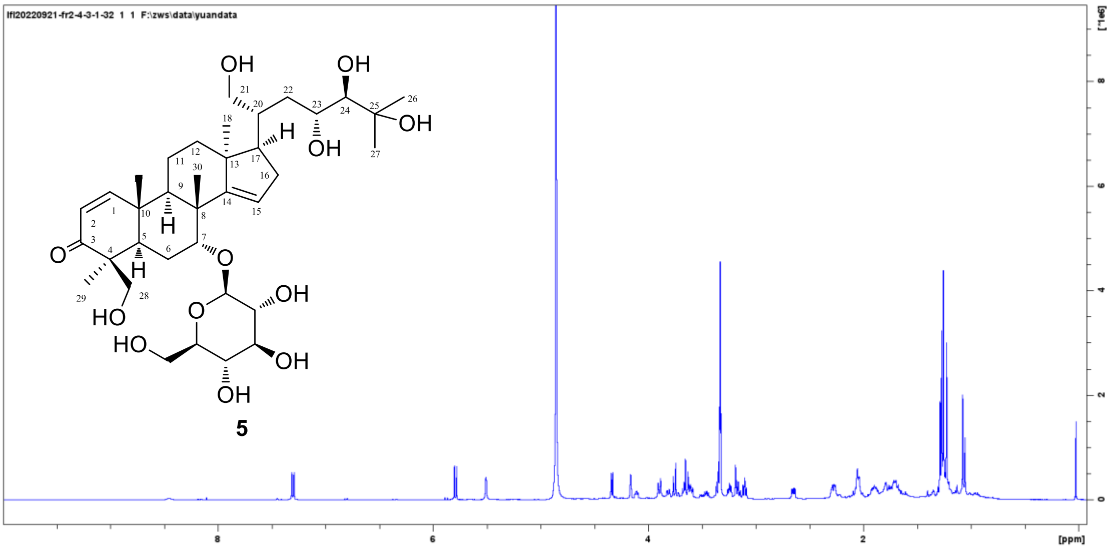

Figure S45. ^{1}H NMR spectrum of munropene E (**5**) in CD_3OD.

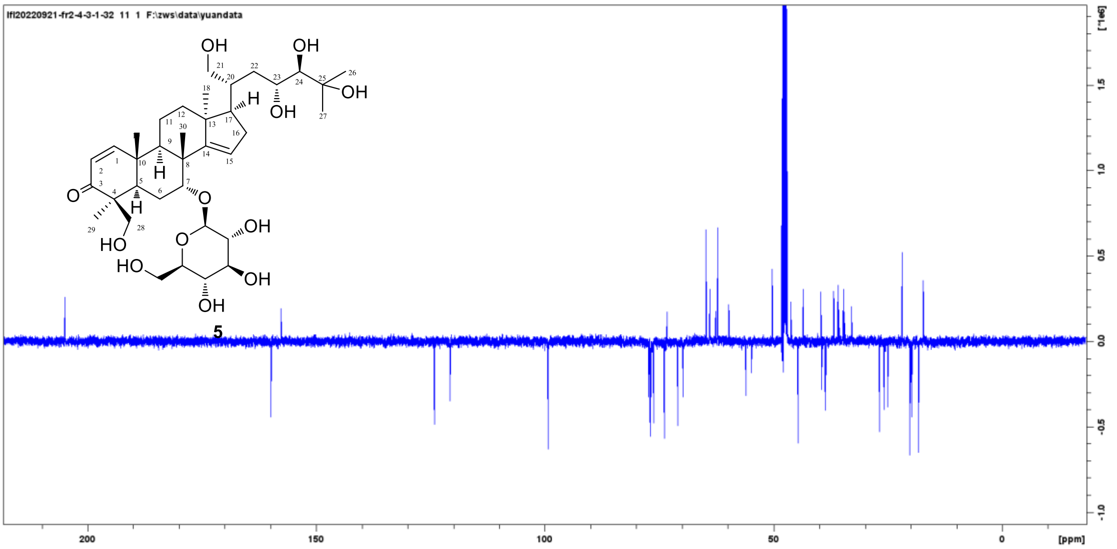

Figure S46. ^{13}C NMR spectrum of munropene E (**5**) in CD_3OD.

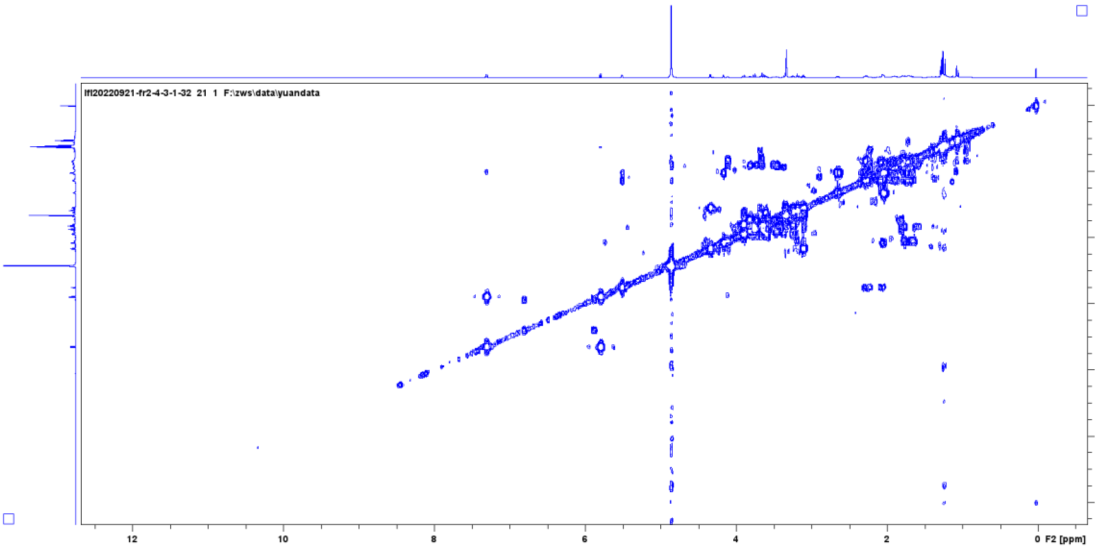

Figure S47. ^{1}H–^{1}H COSY spectrum of munropene E (**5**) in CD_3OD.

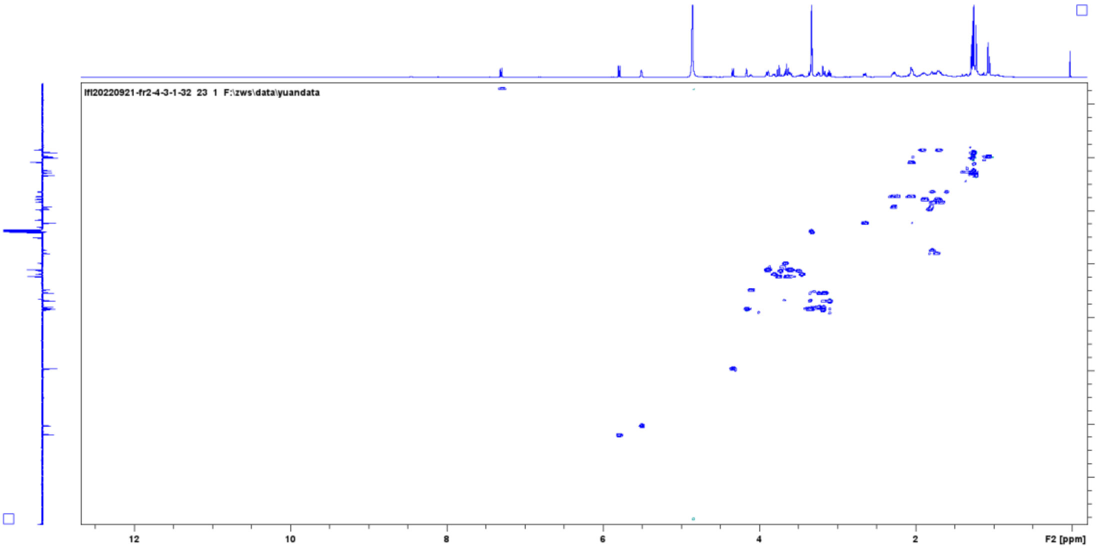

Figure S48. HSQC spectrum of munropene E (**5**) in CD_3OD.

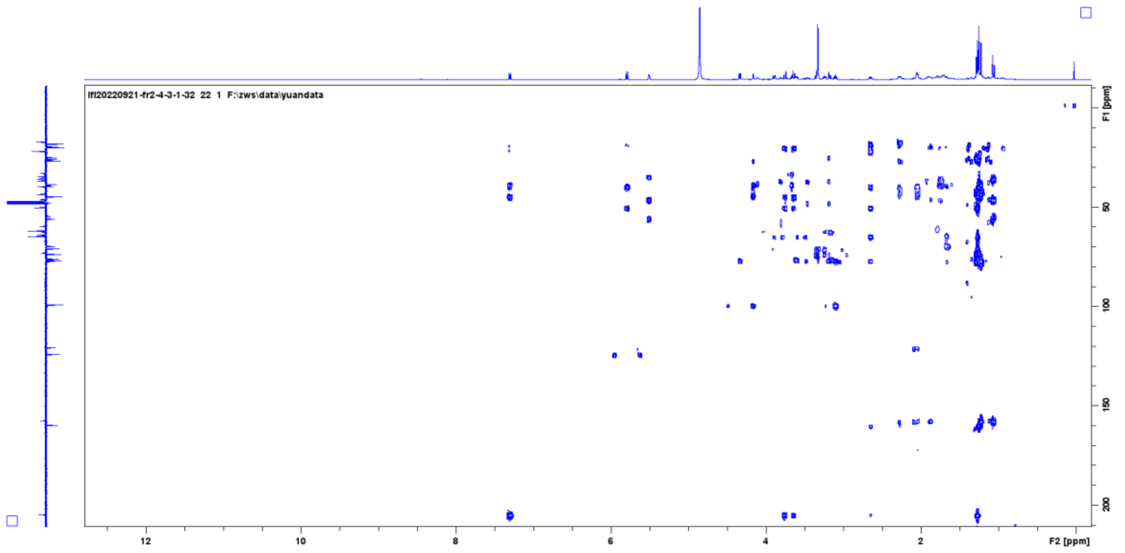

Figure S49. HMBC spectrum of munropene E (**5**) in CD_3OD.

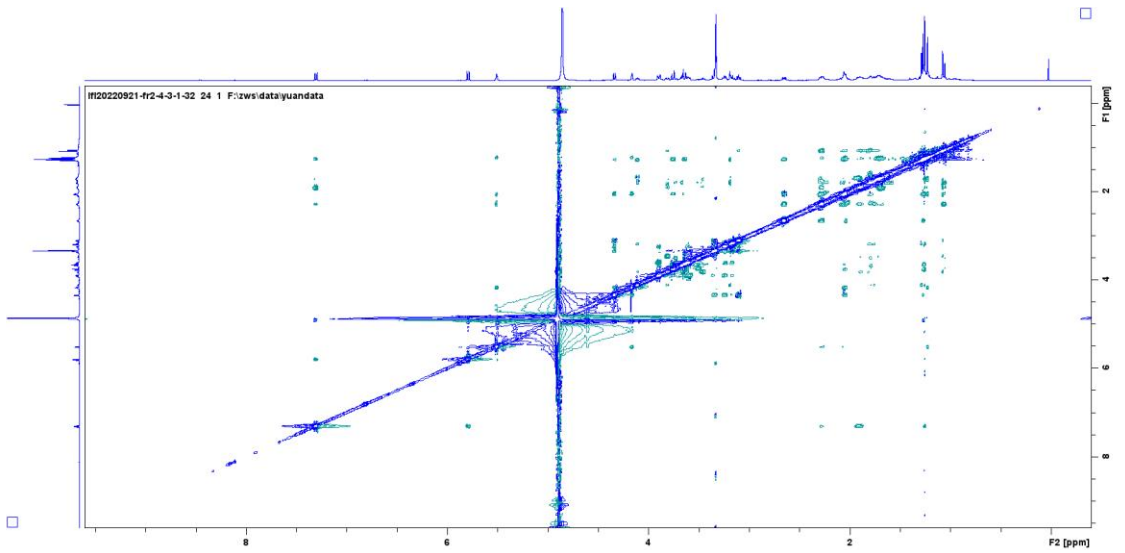

Figure S50. ROESY spectrum of munropene E (**5**) in CD_3OD.

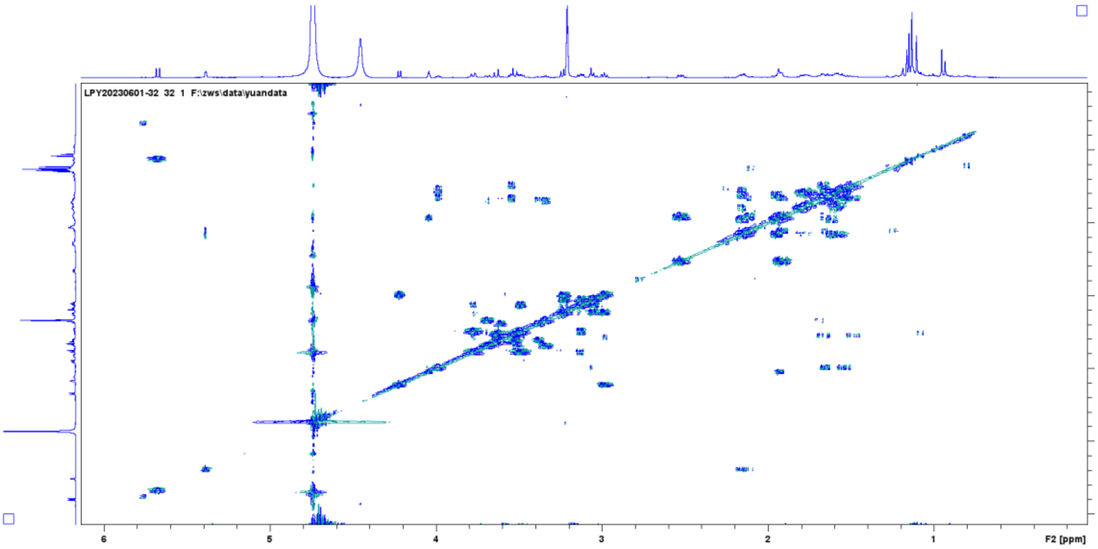

Figure S51. HETLOC spectrum of munropene E (**5**) in CD_3OD.

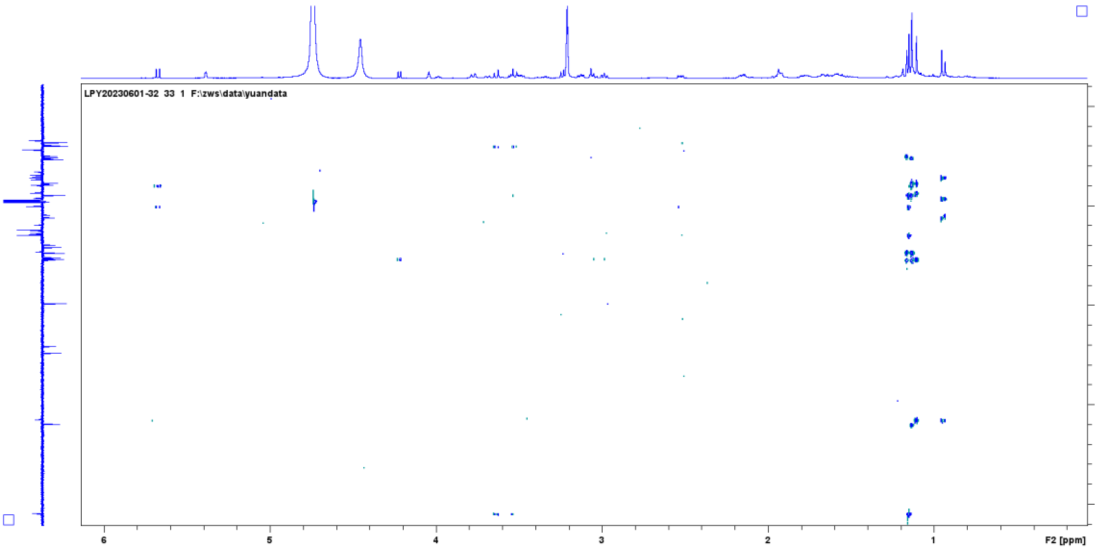

Figure S52. PS-HMBC spectrum of munropene E (**5**) in CD_3OD.

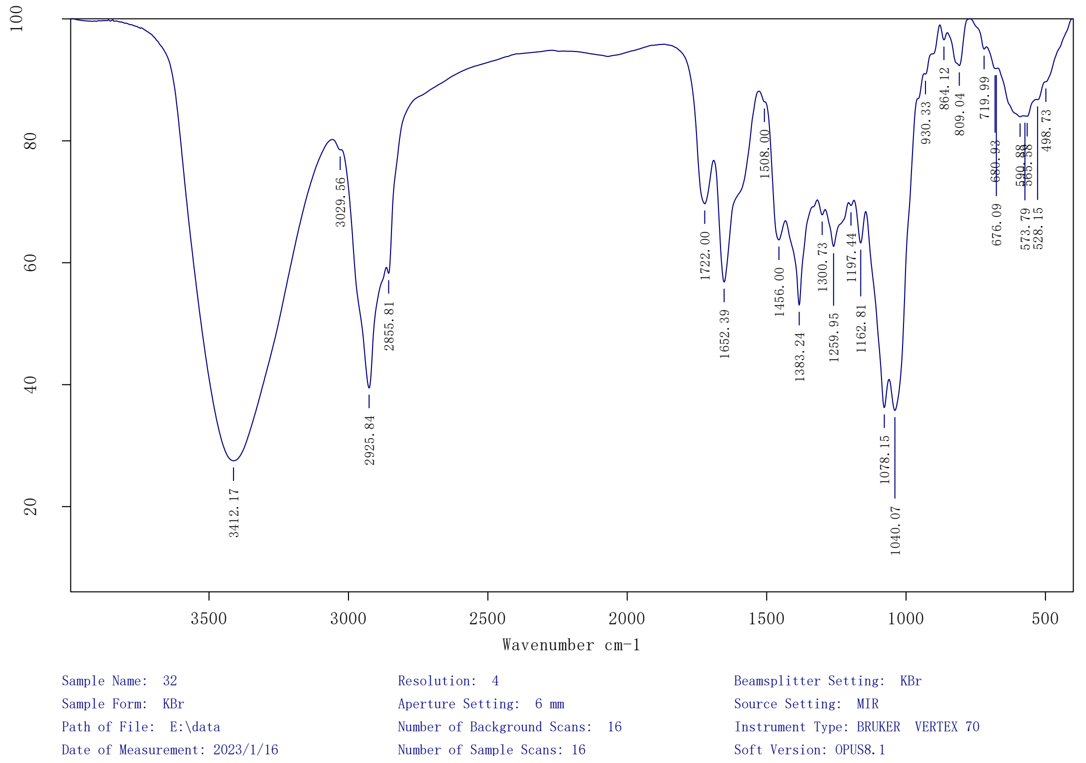

Figure S53. IR spectrum of munropene E (**5**).

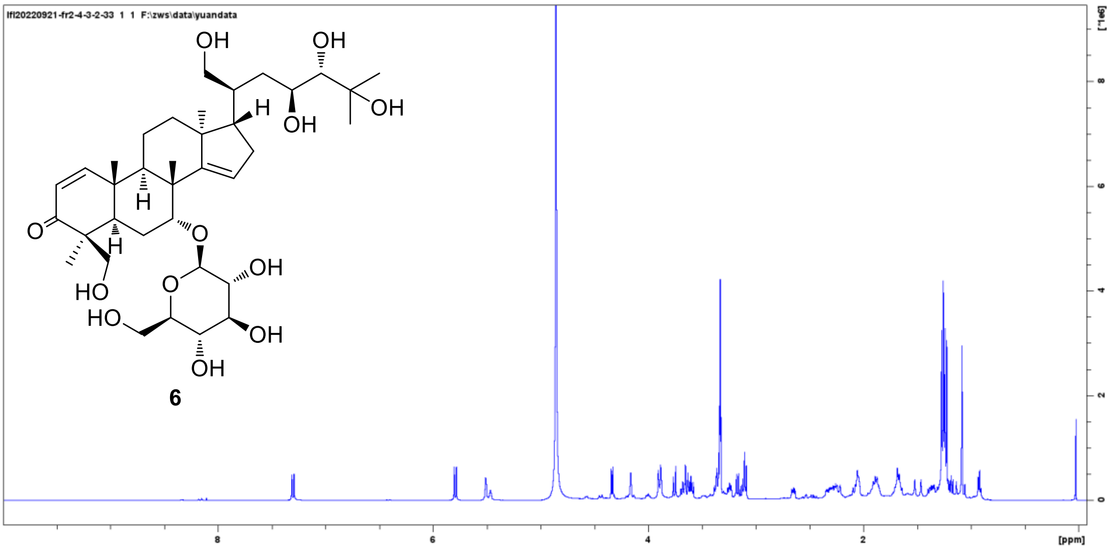

Figure S54. ^{1}H NMR spectrum of munropene F (**6**) in CD_3OD.

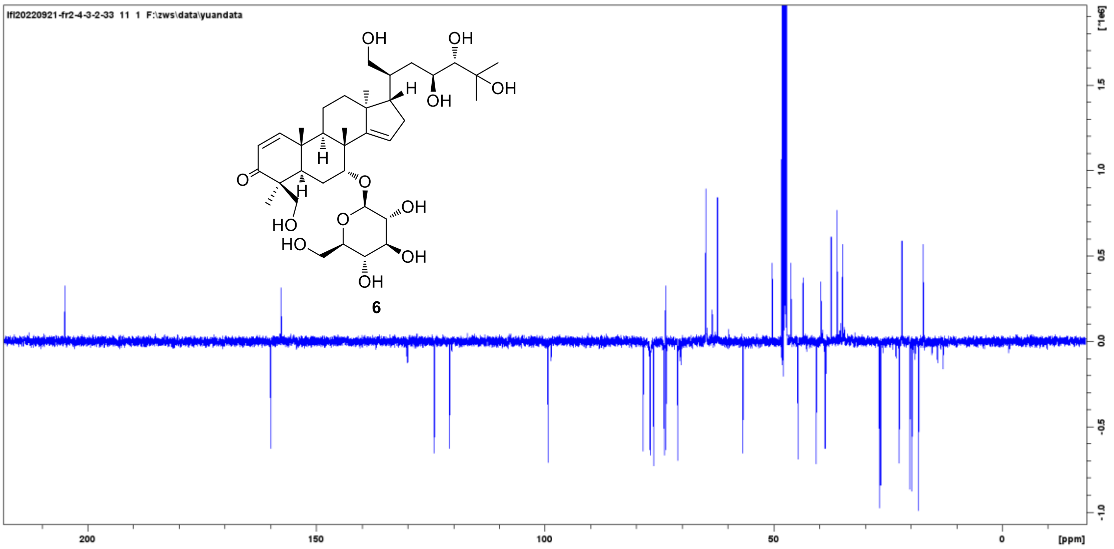

Figure S55. ^{13}C NMR spectrum of munropene F (**6**) in CD_3OD.

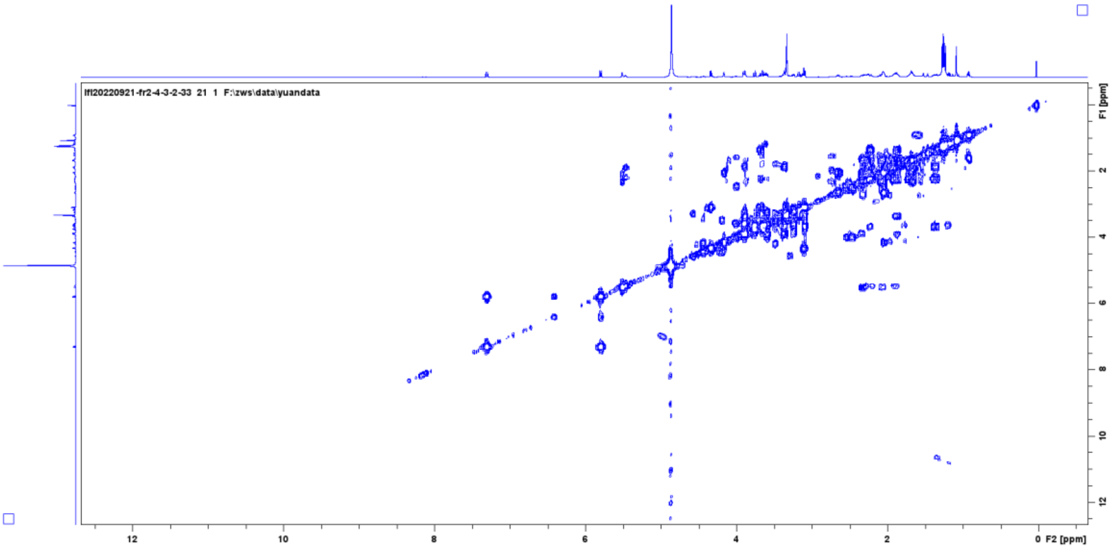

Figure S56. ^{1}H–^{1}H COSY spectrum of munropene F (**6**) in CD_3OD.

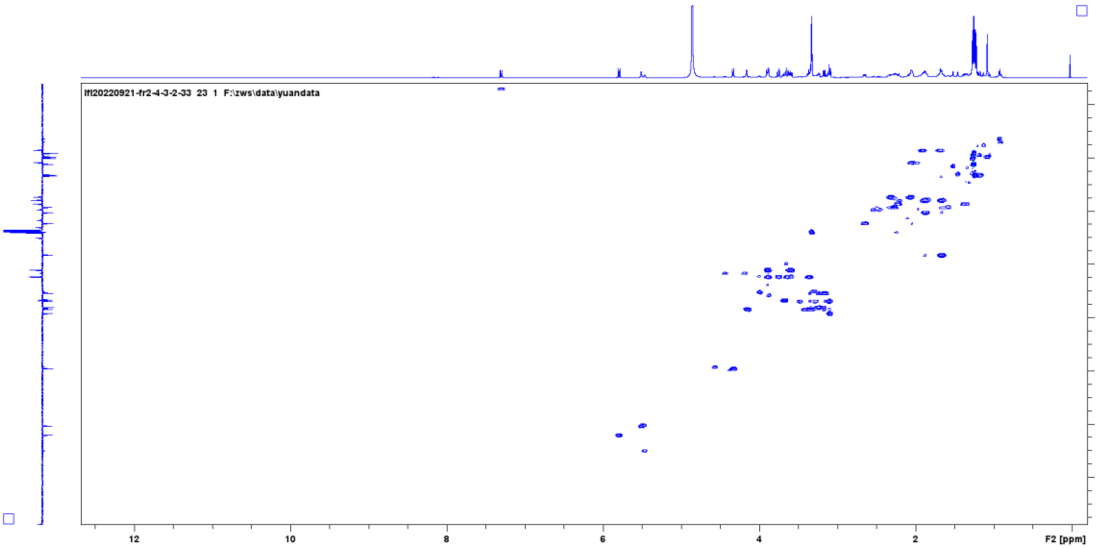

Figure S57. HSQC spectrum of munropene F (**6**) in CD_3OD.

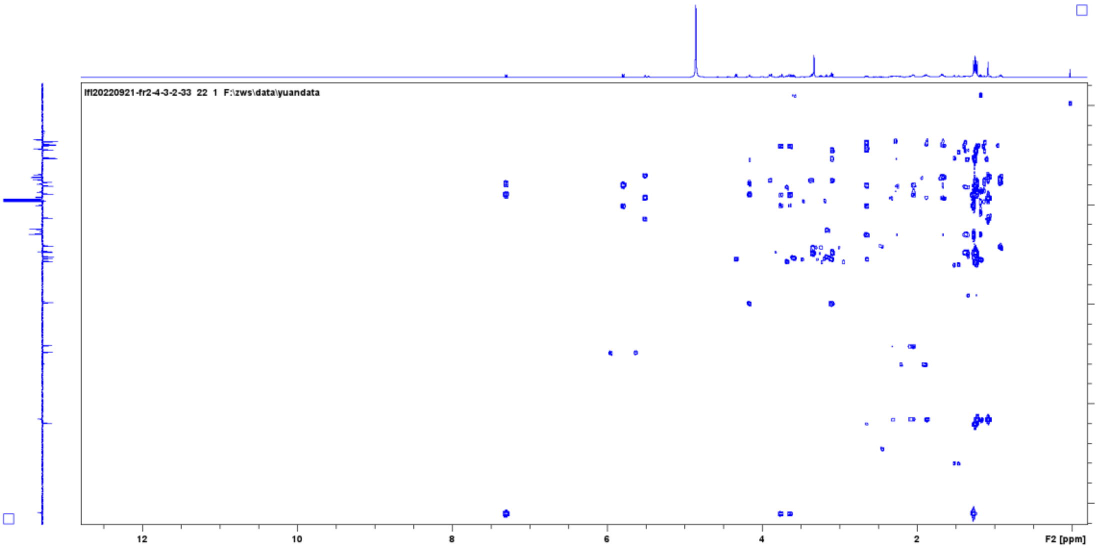

Figure S58. HMBC spectrum of munropene F (**6**) in CD_3OD.

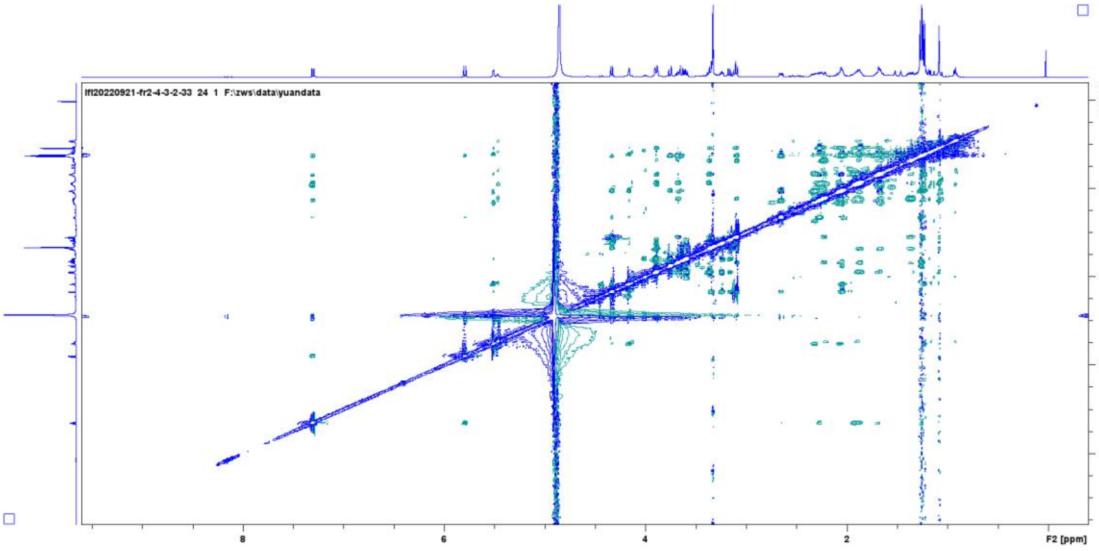

Figure S59. ROESY spectrum of munropene F (**6**) in CD_3OD.

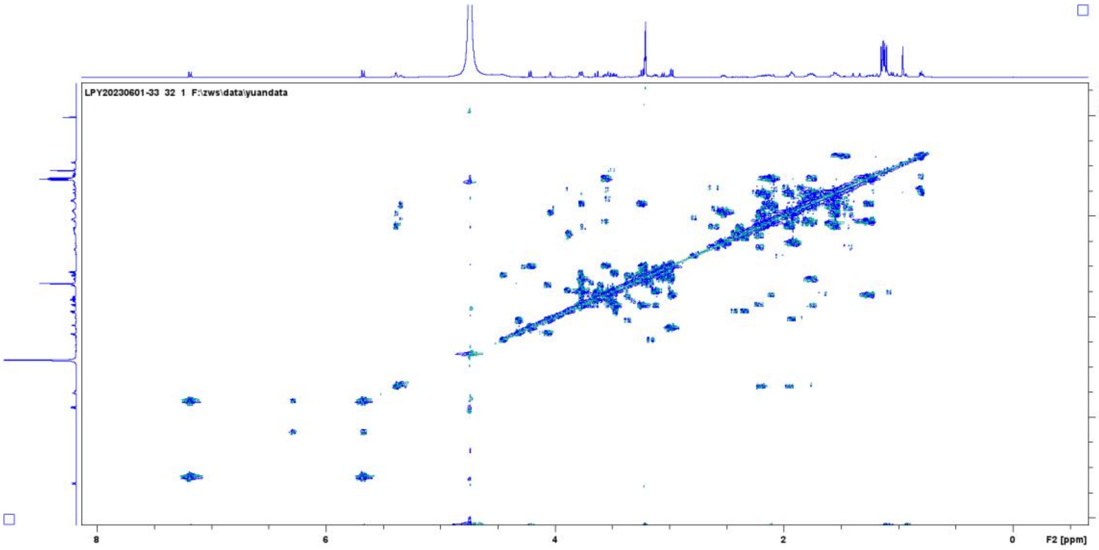

Figure S60. HETLOC spectrum of munropene F (**6**) in CD_3OD.

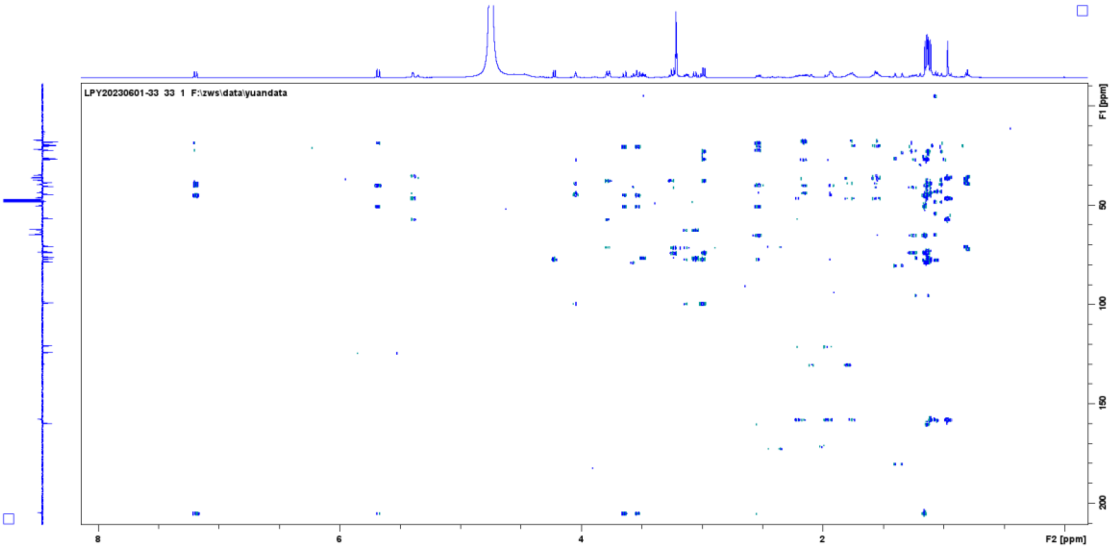

Figure S61. PS-HMBC spectrum of munropene F (**6**) in CD3OD.

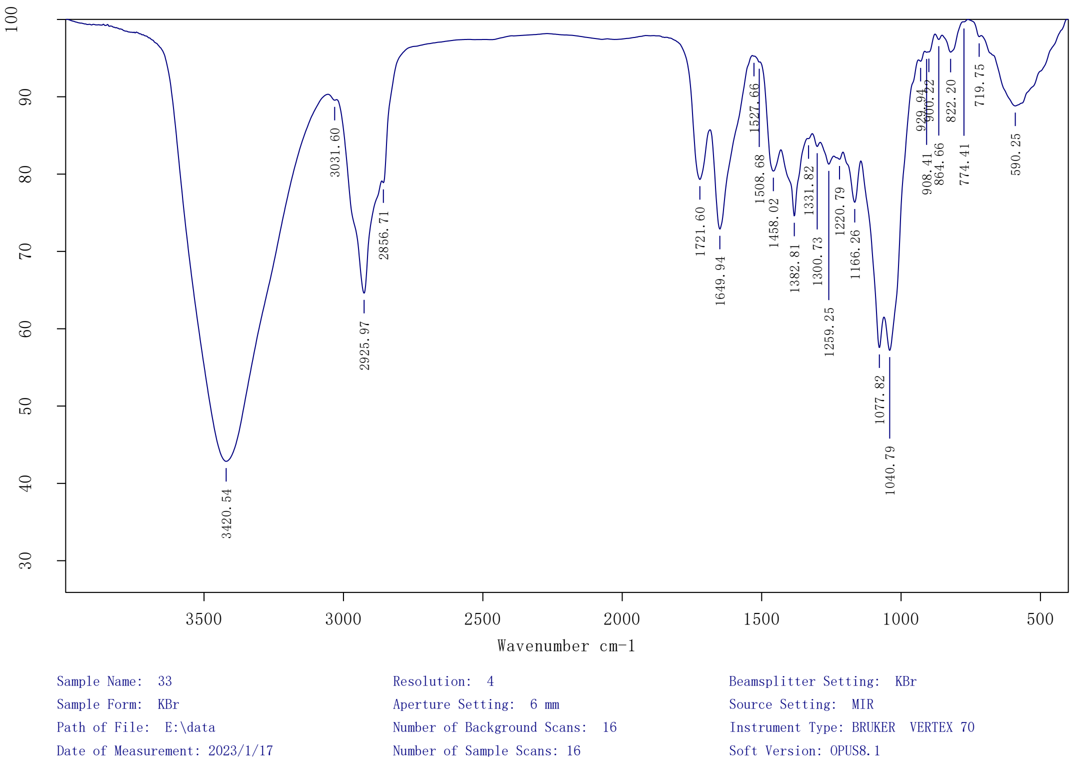

Figure S62. IR spectrum of munropene F (**6**).
